# A Genome-Wide Association Study of Total Serum and Mite-Specific IgEs in Asthma Patients

**DOI:** 10.1371/journal.pone.0071958

**Published:** 2013-08-13

**Authors:** Jeong-Hyun Kim, Hyun Sub Cheong, Jong Sook Park, An-Soo Jang, Soo-Taek Uh, Yong-Hoon Kim, Mi-Kyeong Kim, Inseon S. Choi, Sang Heon Cho, Byoung Whui Choi, Joon Seol Bae, Choon-Sik Park, Hyoung Doo Shin

**Affiliations:** 1 Department of Life Science, Sogang University, Seoul, Republic of Korea; 2 Department of Genetic Epidemiology, SNP Genetics Inc., Seoul, Republic of Korea; 3 Division of Allergy and Respiratory Medicine, Soonchunhyang University Bucheon Hospital, Bucheon, Republic of Korea; 4 Division of Allergy and Respiratory Disease, Soonchunhyang University Cheonan Hospital, Cheonan, Republic of Korea; 5 Division of Internal Medicine, Chungbuk National University, Cheongju, Republic of Korea; 6 Department of Allergy, Chonnam National University, Gwangju, Republic of Korea; 7 Department of Internal Medicine and Institute of Allergy and Clinical Immunology, Seoul National University, Seoul, Republic of Korea; 8 Department of Internal Medicine, Chung-Ang University Yongsan Hospital, Seoul, Republic of Korea; 9 Research Institute for Basic Science, Sogang University, Seoul, Republic of Korea; National Institute of Environmental Health Sciences, United States of America

## Abstract

Immunoglobulin E (IgE) is one of the central players in asthma and allergic diseases. Although the serum IgE level, a useful endophenotype, is generally increased in patients with asthma, genetic factors influencing IgE regulation in asthma are still not fully understood. To identify the genetic variations associated with total serum and mite-specific IgEs in asthmatics, a genome-wide association study (GWAS) of 657,366 single nucleotide polymorphisms (SNPs) was performed in 877 Korean asthmatics. This study found that several new genes might be associated with total IgE in asthmatics, such as *CRIM1* (rs848512, *P* = 1.18×10^−6^; rs711254, *P* = 6.73×10^−6^), *ZNF71* (rs10404342, *P* = 7.60×10^−6^), *TLN1* (rs4879926, *P* = 7.74×10^−6^), and *SYNPO2* (rs1472066, *P* = 8.36×10^−6^; rs1038770, *P* = 8.66×10^−6^). Regarding the association of specific IgE to house dust mites, it was observed that intergenic SNPs nearby to *OPRK1* and *LOC730217* might be associated with *Dermatophagoides pteronyssinus* (D.p.) and *Dermatophagoides farinae* (D.f.) in asthmatics, respectively. In further pathway analysis, the phosphatidylinositol signaling system and adherens junction pathways were estimated to play a role in the regulation of total IgE levels in asthma. Although functional evaluations and replications of these results in other populations are needed, this GWAS of serum IgE in asthmatics could facilitate improved understanding of the role of the newly identified genetic variants in asthma and its related phenotypes.

## Introduction

Asthma, a chronic inflammatory respiratory disease, is characterized by bronchial hyperresponsiveness. Asthma and its related illnesses are complex diseases resulting from interactions among multiple genetic factors as well as environmental components [1]. Despite recent advancements in our knowledge of asthma genetics, the need for a comprehensive etiology of asthma and its related phenotypes still remains. At the same time, it is generally known that patients with asthma show an increase in levels of serum immunoglobulin E (IgE), a closely related endophenotype of asthma [2], [3]. Furthermore, several loci, such as the *CTLA4* and *C11orf30-LRRC32* regions, have been shown to be associated with total serum IgE levels in patients with asthma [2], [4]. Two genome-wide association studies (GWASs) on total IgE in four population-based cohorts [5] and in subjects combined with asthmatics and controls [6] have identified *FCER1A* as a novel susceptibility locus. Furthermore, two other recent GWASs have found additional genes (such as *IL13* and *HLA-DQB1*) that are associated with the total serum IgE [7], [8]. However, given that asthma is a complex disease and that the nature of the associations between genetic variations and levels of IgE in asthmatics is not yet fully understood, this study aims to identify additional risk loci for the elevation of total serum IgE in asthmatics.

IgE is a class of antibody that plays an essential role in immediate hypersensitivity response, which is the hallmark of allergic diseases including asthma [9], [10]. Omalizumab, a humanized antibody drug against IgE, is clinically effective in patients with moderate to severe, persistent allergic asthma [11], indicating that IgE has an important role in the development of allergies and allergic responses. Several IgE-influencing genes that are significantly associated with asthma have been reported [4], [5], [12], [13]. In the case of *interleukin 4* (*IL4*) and *IL13*, which stimulate B cells to produce IgE, genetic variants have been determined to be significantly associated with serum IgE in childhood asthma and atopy [12], [14]. However, more recent studies have suggested that new candidate genes, such as *DPP10* and the *C11orf30*-*LRRC32* region, may affect IgE levels in asthmatics [2], [15]. Therefore, considering that asthma is a heterogeneous and very complex disease, a more specific investigation using a GWAS of IgE in asthma cohorts could increase understanding of the pathogenesis of the disease, and may provide a new strategy for its control.

It has been reported that asthma severity is positively correlated with serum concentration of both total IgE and specific IgE to *Dermatophagoides pteronyssinus* (D.p.) [16]. In addition, house dust mites (HDMs), D.p. and *Dermatophagoides farinae* (D.f.), are generally considered among the most implicated asthma triggers associated with blood allergen-specific IgEs [17]. Furthermore, levels of allergen-specific IgE against D.p. and D.f. in serum have been found to be significantly increased in the bronchial allergen challenge with HDM accompanied by enhanced Th2-cytokine production [18]. Therefore, based on these facts, this study has performed a GWAS for each of allergen-specific IgEs (D.p. and D.f.).

## Subjects and Methods

### Study Subjects

A total of 877 asthma patients were recruited from Soonchunhyang University, Chungbuk National University, Chonnam National University, Seoul National University, and Chung-Ang University in Korea. All subjects provided written informed consent. In the case of children patients, their parents provided written informed consent before the study commenced. The study protocols were approved by an Institutional Review Board of the Soonchunhyang University Bucheon Hospital, a member of the National Biobank of Korea (IRB No. SCHBC_IRB_05_02). All patients met the criteria for clinical symptoms in accordance with the definition of asthma in the Global Initiative for Asthma (GINA) guidelines. All patients had airway reversibility, as documented by a positive bronchodilator response of >15% increase in forced expiratory volume in one second (FEV_1_) and/or airway hyperreactivity to <10 mg/mL of PC_20_ methacholine. Twenty-four common inhalant allergens, including dust mites (D.p. and D.f.), car fur, dog fur, cockroaches, grass, tree, pollens, ragweed, and the aspergillus species (Bencard Co., Brentford, UK), were used for the skin-prick test. Atopy was defined as having a wheal reaction to an allergen that was equal to or greater than that to histamine (1 mg/ml), or that was 3 mm in diameter.

Total IgE and specific IgE to D.p. and D.f. were measured using the UniCAP system (Pharmacia Diagnostics, Uppsala, Sweden). UniCAP-specific IgE calibrators were used for the determination of specific IgE antibodies. Specific IgE (D.p and D.f.) was classified as 0-6, based on the semi-quantitatively expressed concentrations (Table S1). For UniCAP allergen, 0.35 kU/l was used as a cut-off value (value <0.35 kU/l was a negative result, whereas value >0.35 kU/l was a positive result). A positive result indicates presence of specific IgE antibodies to one or more of the allergens coupled to UniCAP allergens [19].

### Genome-wide Genotyping

Genome-wide SNP genotyping of asthma subjects was performed using approximately 200 ng of genomic DNA from the subjects’ peripheral blood lymphocytes on Illumina’s Human660W-Quad BeadChip® (Illumina, San Diego, CA, USA), according to the manufacturer’s protocol. Quality controls of call rate (>98%) and high minor allele frequency (MAF >0.05) were measured. In addition, visual inspection of the genotype cluster image was performed for the SNPs with deviation from Hardy-Weinberg equilibrium (HWE) and with an association *P*-value <0.001. SNPs in chromosome X of males were excluded from HWE analysis. Although there were significantly deviated SNPs from HWE (as measured by an asymptotic test) in asthmatics, these SNPs were included in the association analysis in order not to miss any SNPs underlying both asthma and total or specific IgEs (i.e., pleiotropy). X-chromosomal markers were also included in the association study. For the additive genetic model among males, genotypes of X chromosome were coded as “0” (homozygous for the major allele) or “2” (homozygous for the minor allele). A total of 70,601 SNPs were excluded, and 442,098 SNP markers were examined for further association analysis.

### Statistics

All subjects were of Korean ethnicity. Using the principal component analysis (PCA) for population stratification [20], the genomic control inflation factor (λ, calculated by dividing median χ^2^ statistics by 0.456) was computed as 1.003, indicating no significant deviation of the population stratification. For genome-wide evaluation, associations of genotype distributions were calculated using linear regression analysis for total IgE (IU/ml, log_10_-transformed) association and logistic analysis for specific IgE association, adjusted for age (continuous value), sex (male = 0, female = 1), and smoking status (non-smoker = 0, ex-smoker = 1, current smoker = 2) as covariates, using HelixTree® software (Golden Helix, Bozeman, MT, USA). Statistical power analysis of linear regression for total IgE was obtained from a two-tailed *t*-test; case-control analysis for specific IgE was obtained from the CaTS power calculator [21], using heritability estimates for total IgE (0.49), specific IgE against D.p. (0.24), and specific IgE against D.f. (0.25) [22].

Statistical test used in the Pathway Express (http://vortex.cs.wayne.edu/projects.htm) is represented by the corrected gamma *P*-value, as the *P*-value provided by an impact analysis calculated as the sum of the statistically significant number of genes showing biological meanings in the given pathway [23]. In our pathway analysis, the list of gene symbols, which include top SNPs, has been inputted as instructed in the pathway analysis program.

## Results

Demographic characteristics of study subjects are summarized in Table 1. A total of 877 Korean asthmatics were involved in the GWAS, including two subgroups for specific IgEs (D.p. and D.f.): 329 (37.5%) D.p.-positive vs. 548 (62.5%) D.p.-negative and 404 (46.1%) D.f.-positive vs. 473 (53.9%) D.f.-negative. Female-male ratio was ∼1.8∶1 for asthmatics, as was expected in light of general gender differences in adult asthma according to the Global Allergy and Asthma European Network (GA2LEN). Also as expected, atopy was more prevalent in specific IgE-positive groups compared to the negative groups. Specific IgE-positive asthmatics showed higher total IgE levels compared to those of specific IgE-negative patients (*P*<0.001, Table 1).

**Table 1:** Clinical profiles of study subjects.

		Asthmatics for specific IgE (D.p.)		Asthmatics for specific IgE (D.f.)	
Clinical profile	Asthmatics	Positive	Negative	Positive	Negative
Number of subjects (n)	877	329	548	404	473
Age [year, mean (range)]	45.3 (10.8–77.5)	39.0 (10.8–77.1)	49.1 (14.1–77.5)	41.1 (10.8–77.1)	49.0 (16.1–77.5)
Sex (n, male/female)	320/557	147/182	173/375	190/214	130/343
Smoking (n, No/Yes/Ex)	619/118/140	217/59/53	402/59/87	254/77/73	365/41/67
Atopy (n, No/Yes)	397/480	23/306	374/174	48/356	349/124
Log_10_[Total IgE (IU/ml)]	2.18±0.65	2.50±0.51*	1.98±0.64*	2.47±0.56*	1.93±0.61*

Ex indicates ex-smoker.

D.p., Dermatophagoides pteronyssinus; D.f., Dermatophagoides farinae.

* *P*<0.001.

### Total IgE Association in Asthmatics

A total of 442,117 SNPs were passed through strict quality-control measures (call rate >98%, MAF >0.05). First, we tested association with total serum IgE levels in asthmatics using linear regression analysis corrected for age, gender, and smoking status as covariates. In the case of genetic homogeneity of the study subjects, the measured genomic control inflation factor was λ = 1.003, indicating no inflation of the type I error. The quantile-quantile (Q–Q) plot for the association test with total IgE showed that distributions between observed and expected *P*-values were deviated in the extreme tail (Figure S1A).

**Figure 1:**
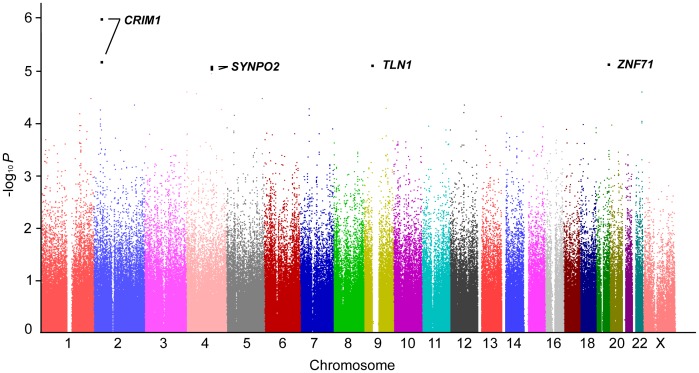
Graphical summary (Manhattan plot) presenting *P*-values for the association with total serum IgE in asthma patients. The y-axis represents -log_10_ *P* (linear regression analysis) from 442,117 SNPs in 877 patients with asthma, correcting for age, gender, and smoking status as covariates; the x-axis indicates its physical position on successive chromosomes.

**Figure 2:**
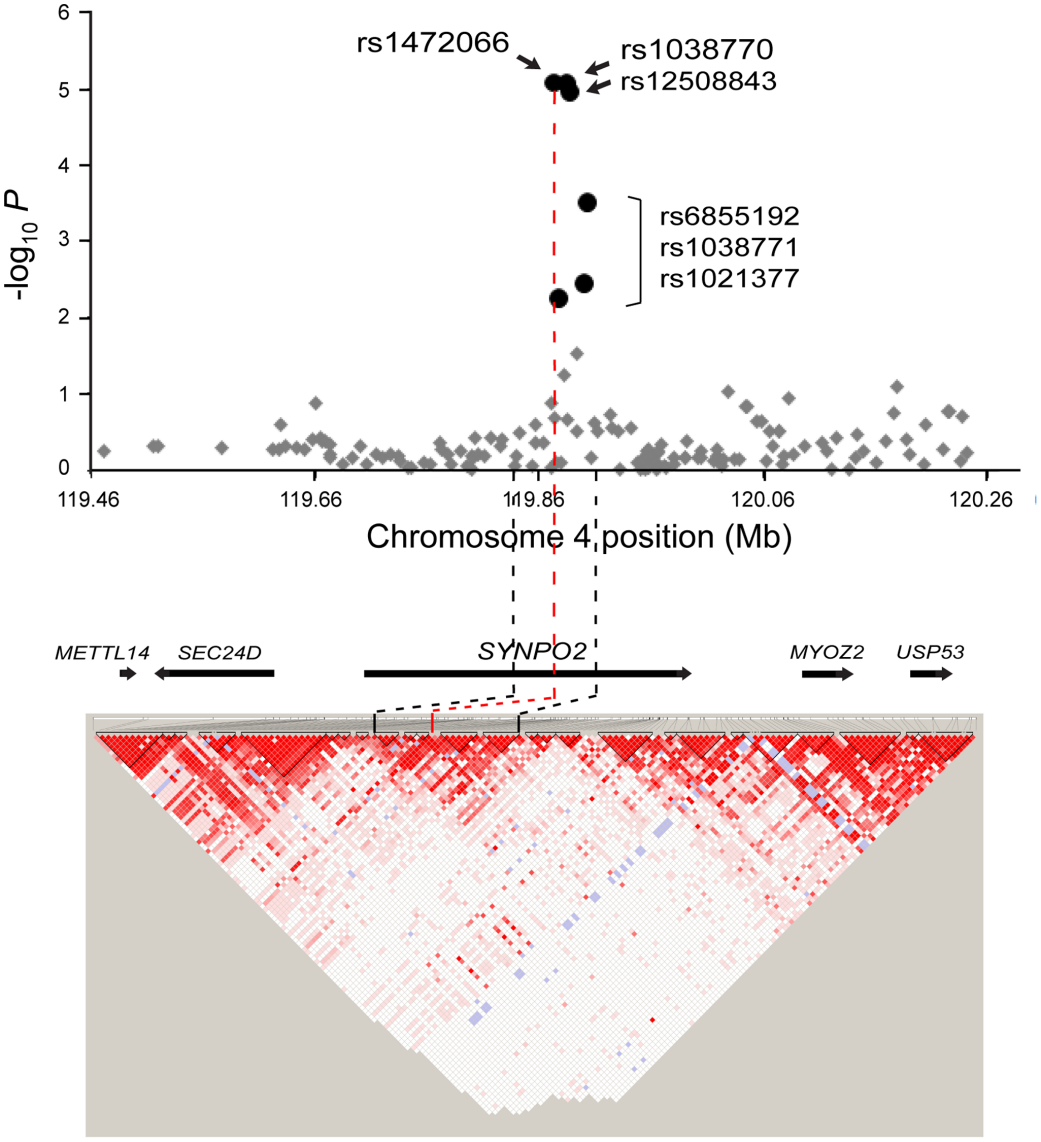
Regional association plot and LD of *SYNPO2* for the total IgE in asthmatics. GWAS associations (-log_10_ *P*) of SNPs across approximately 768 kb region around *SYNPO4* in the chromosome 4q26 are shown. Relatively strong associations are shown as large black circles; relatively less significances as small gray diamonds.

Although no individual SNPs reached a genome-wide significant level (Bonferroni-corrected significance), top signals (*P*<1.0×10^−5^, Table 2 and Table S2) identified several new loci in the intronic region of genes, such as *CRIM1* (rs848512, *P* = 1.18×10^−6^; rs711254, *P* = 6.73×10^−6^), *ZNF71* (rs10404342, *P* = 7.60×10^−6^), *TLN1* (rs4879926, *P* = 7.74×10^−6^), and *SYNPO2* (rs1472066, *P* = 8.36×10^−6^; rs1038770, *P* = 8.66×10^−6^), as susceptibility markers for total IgE levels in asthmatics (Figure 1). In further regional association of each new locus associated with IgE, six SNPs of *SYNPO2* in the chromosome 4q26 (Figure 2) showed relatively strong association signals (*P* = 8.36×10^−6^ for rs1472066 to *P* = 0.0056 for rs1021377) when compared to other top signals (Figure S2, S3, S4). In the case of linkage disequilibrium (LD) analysis, it was revealed that *SYNPO2* or its SNPs that showed significant associations with total IgE were likely to not be in LD with nearby genes (*r^2^*≤0.243, Figure 2).

**Table 2:** Top 6 SNPs (*P*<1.0×10^−5^) associated with total IgE of asthmatics in the GWAS.

								Genotype**			
SNP ID	Chr	Gene	Location	Variation	MAF	LD (coefficient *r^2^*)	HWE*	M/M†	M/m†	m/m†	*P*-value
rs848512	2	*CRIM1*	Intron	C>T	0.078	0.925 (rs848512- rs711254)	0.283	744 (2.22)	130 (1.93)	3 (1.89)	1.18×10^−6^
rs711254	2	*CRIM1*	Intron	C>T	0.083		0.373	735 (2.22)	137 (1.97)	4 (1.76)	6.73×10^−6^
rs10404342	19	*ZNF71*	Intron	A>C	0.399		0.748	315 (2.05)	425 (2.23)	137 (2.33)	7.60×10^−6^
rs4879926	9	*TLN1*	Intron	T>C	0.072		0.070	751 (2.22)	125 (1.96)	1 (2.3)	7.74×10^−6^
rs1472066	4	*SYNPO2*	Intron	A>G	0.107	0.581(rs1472066−rs1038770)	0.744	700 (2.23)	166 (1.99)	11 (1.81)	8.36×10^−6^
rs1038770	4	*SYNPO2*	Intron	G>A	0.116		0.805	685 (2.23)	181 (2.02)	11 (1.6)	8.66×10^−6^

Association analyses were adjusted by age, sex, and smoking status as covariates.

* *P*-value of Hardy-Weinberg equilibrium (HWE).

** Genotype represents number of subjects (mean of log[Total IgE (IU/ml)]).

† M/M, M/m, and m/m indicate homozygote for common allele, heterozygote, and homozygote for rare allele, respectively.

Chr, chromosome; MAF, minor allele frequency.

### Specific IgE (D.p. and D.f.) Associations in Asthmatics

In further association analyses with specific IgE to house dust mites (Figure S5), along with Q–Q plots for the association test with specific IgE against D.p. and D.f. (Figure S1B and S1C), intergenic SNPs nearby to *OPRK1* (rs1425902, *P* = 1.44×10^−6^, Table S3) and *LOC730217* (rs10142119, *P* = 1.86×10^−7^; rs1456988, *P* = 1.11×10^−6^; rs12590389, *P* = 2.47×10^−6^, Table S4, coefficient *r^2^* among these three SNPs = 0.515–0.757) were detected as the most significant polymorphisms associated with the specific IgEs of D.p. and D.f. in asthmatics, respectively.

### Pathway Analysis

To better understand the biological process and molecular function involved in total serum IgE in asthma, the signaling pathways were estimated using Pathway Express (http://vortex.cs.wayne.edu/projects.htm). Among the significantly associated SNPs with total IgE (*P*<0.001), only the SNPs in the gene regions were analyzed. The phosphatidylinositol signaling system (*P* = 2.89×10^−18^) and adherens junction (*P* = 1.57×10^−13^) pathways were estimated to play a role in the regulation of total IgE levels in asthmatics (Table S5).

## Discussion

IgE is considered an important target in the treatment of asthma. Furthermore, anti-IgE therapy, i.e., using a humanized antibody drug of Omalizumab against IgE, has been demonstrated to be efficacious for the management of asthma as well as allergic diseases [11], [24]. Asthma susceptibility is generally classified into four main mechanisms as follows: (1) innate immunity and immunoregulation, (2) T_H_2-cell differentiation and effector function, (3) epithelial cells, and (4) lung function. Although IgE and its binding to FcεRI (high-affinity Fc receptor for IgE) regulates the activation of mast cells and basophils mainly in the first and second mechanisms, IgE is associated with a more complicated network of proteins [10], [25]. Therefore, together with the identification of new potential risk factors, our GWAS of total serum IgE and mite-specific IgE in asthmatics may provide additional supporting information about genetic associations related to IgE function in asthma and related diseases.

In this study, no individual SNPs reached a genome-wide significance level (Bonferroni-corrected significance), which is a limitation of this study. According to the power calculation, this failure to reach a genome-wide signal might be due to an underpowered study (in particular, statistical powers of 44.5% in total IgE analysis and 62.0% in specific IgE against D.f., Table S6), because of insufficient sample size and lack of replication. In order to address this limitation, further replications in larger cohorts are needed. On the other hand, being that asthma is a complex disease, we also cannot rule out the possibility that confounders with small to modest effects may contribute to the regulation of total IgE in asthma [26]. Our results identified new potential genes associated with total IgE in asthmatics, such as *CRIM1*, *ZNF71*, *TLN1*, and *SYNPO2*. Recently, *SYNPO2* has been reported to be significantly associated with airway hyperresponsiveness in patients with asthma [27]. In addition, in our further in silico analysis using the Human Splicing Finder (http://www.umd.be/HSF/) [28], rs4879926 of *TLN1*, rs1472066 and rs1038770 of *SYNPO2* were predicted as potential branch point (BP) sites for alternative splicing (Figure S6), revealing that these variations might have a possibility to produce an isoform of the gene. These results suggest that genetic variants of the newly discovered genes, in particular *SYNPO2*, could play a role in IgE regulation.

Recent results from GWASs on total serum IgE levels have identified associated genes, including *RAD50*, *STAT6*, *FCER1A*, *IL13*, and *HLA-DQB1* [5]–[8]. When genetic associations of the identified SNPs from previous GWASs were compared with those from this GWAS, most were not replicated in our Korean asthmatic subjects (Table S7). Instead, this study identified additional candidate genes (*CRIM1*, *ZNF71*, *TLN1*, *SYNPO2*, etc.) for total serum IgE levels in asthma patients. Among the several well-known IgE-influencing genes (*IL4*, *IL13*, *CD28*, *C11orf30*-*LRRC32* region, etc.) [2], [4], [12], [15], [29], this study confirmed a significant association between *CD28* SNPs and total IgE in asthmatics (minimum *P* = 0.001, data not shown). Serum total IgE levels have been elucidated to be correlated with CD28, an important regulator of T-cell activation and subsequent IgE production [30], [31]. In this study, differences in the results between present and previous GWASs might be attributed to population and/or ethnic differences, limitation and mismatch of the tested SNP markers (albeit only SNPs in the *C11orf30*-*LRRC32* region were matched with those of this GWAS), and a diversity of clinical phenotypes among asthma patients.

As other potential genes that are not as well-known but that potentially affect IgE levels, SNPs of *NPSR1* and *IRAK3* were also observed to be associated with total IgE among asthmatics in this study (Table S8). SNP-tagged haplotypes of *neuropeptide S receptor 1* (*NPSR1*; also known as G-protein-coupled receptor for asthma susceptibility, *GPRA* or *GPRA154*) have been found to be associated with increased serum IgE levels or asthma, and have been functionally evaluated to be distinctively distributed between protein isoforms in bronchial biopsies from healthy and asthmatic subjects [32]. However, since conflicting results in associations of *NPSR1* and its genetic variations with total IgE level and/or asthma have also been reported [33], [34], further studies are required to determine their effect on the disease trait. In addition, *IRAK3* rs1821777, which showed a nominal signal (Table S8) in the present study, has also been reported to be related to asthma in North Americans (*P* = 0.03) and in Sardinians (*P* = 0.001) of Italy [35], [36]. Two *IRAK3* SNPs (rs2701653 and rs1821777, Table S8) were also observed to be related to atopic asthma in a Spanish population (*P* = 0.034) and in a meta-analysis of Spanish and Sardinian populations (*P* = 0.013) [37]. In light of the potential association of the nonsynonymous variant rs1152888 of *IRAK3* with total IgE in asthmatics (*P* = 0.003, Table S8), further functional evaluations will be valuable in identifying whether *IRAK3* can serve as a new therapeutic target.

Given that a previous study showed the association between total serum IgE and asthma independently of specific IgE levels to mites [38], we performed further analyses to discover factors associated with specific IgE against two major house dust mites (D.p. and D.f.) in asthmatics. Similar to the previous study, totally different profiles of genetic variants between total IgE and mite-specific IgEs (possibly even between specific IgEs against D.p. and D.f.) in asthmatics were observed in the further analyses. These observations suggest that patients with asthma may have allergies to different environmental allergens such as cockroaches and pet allergens [39], [40], as well as house dust mites.

To better understand the biological process and molecular function involved in total serum IgE in asthma, the signaling pathways were estimated using Pathway Express (http://vortex.cs.wayne.edu/projects.htm). The result showed that the phosphatidylinositol signaling system (*P* = 2.89×10^−18^), as supported by previous strong evidence [41], [42], and adherens junction (*P* = 1.57×10^−13^) pathways might play a role in the regulation of total IgE levels in asthmatics (Table S5). In addition, in the Gene Relationships Across Implicated Loci (GRAIL) analysis based on PubMed articles published before December 2006, several genes (*KALRN*, *CNTN5*, *INPP4B*, etc.; *P* = ∼0.01) were shown to have functional connectivity (Table S9). Thus, despite study limitations (lacks of genome-wide significance, replication, and functional evaluation), our GWAS of IgE in asthmatics may provide an outline of the genetic implication for total serum and mite-specific IgEs in asthma and its related phenotypes.

## Supporting Information

Figure S1 **Q-Q plots of total IgE, specific IgE (D.p.), and specific IgE (D.f.).** The observed *P*-value (y-axis) is compared with the expected *P*-value (x-axis, under null distribution) for (A) total IgE, (B) specific IgE (D.p.), and (C) specific IgE (D.f.).
(DOC)

Figure S2 **Regional association plot and LD of** ***CRIM1*** **for the total IgE in asthmatics.** GWAS associations (−log_10_ *P*) of SNPs across approximately 310 kb region around *CRIM1* in the chromosome 2p21 are shown. Relatively strong associations are shown as large black circles; relatively less significances as small gray diamonds.
(DOC)

Figure S3 **Regional association plot and LD of** ***ZNF71*** **for the total IgE in asthmatics.** GWAS associations (−log_10_ *P*) of SNPs across approximately 245 kb region around *ZNF71* in the chromosome 19q13.4 are shown. Relatively strong associations are shown as large black circles; relatively less significances as small gray diamonds.
(DOC)

Figure S4 **Regional association plot and LD of** ***TLN1*** **for the total IgE in asthmatics.** GWAS associations (−log_10_ *P*) of SNPs across approximately 126 kb region around *TLN1* in the chromosome 9p13 are shown. Relatively strong associations are shown as large black circles; relatively less significances as small gray diamonds.
(DOC)

Figure S5 **Manhattan plots of specific IgE against house dust mites (D.p. and D.f.).** Associations for the specific IgE against (A) D.p. and (B) D.f. in asthma patients are obtained by correcting for age, gender, and smoking status as covariates.
(DOC)

Figure S6 **In silico analysis of top 6 SNPs in intron regions.** (A) Potential branch point (BP) sites are predicted by the change between major allele and minor allele using the Human Splicing Finder (http://www.umd.be/HSF/). (B) A schematic plot of alternative splicing by the predicted additional BP site. The picture of consensus sequence is modified from Desmet et al. Nucleic Acids Research 37(9): e67, 2009.
(DOC)

Table S1 **Classification of specific IgE.**
(DOC)

Table S2 **Top 100 SNPs associated with total IgE of asthmatics in the GWAS.**
(DOC)

Table S3 **Top 100 SNPs associated with specific IgE for D.p. in the GWAS.**
(DOC)

Table S4 **Top 100 SNPs associated with specific IgE for D.f. in the GWAS.**
(DOC)

Table S5 **Pathway analysis for significantly associated SNPs (** ***P*** **<0.001), only in the gene regions from the results of GWAS, on total IgE in asthmatics.**
(DOC)

Table S6 **Statistical power for the analysis in each subgroup of study subjects.**
(DOC)

Table S7 **Genetic associations from this GWAS compared to the previously identified SNPs from other GWASs.**
(DOC)

Table S8 **SNPs of other potential genes (but not well-known IgE-influencing genes) associated with total IgE in asthmatics (** ***P*** **<0.01).**
(DOC)

Table S9 **Genes based clustering from GRAIL.**
(DOC)
