# Supplementary figures and images for: A Genome-Wide Association Study of Total Serum and Mite-Specific IgEs in Asthma Patients
Source: PLoS One. 2013 Aug 13;8(8):e71958. doi: 10.1371/journal.pone.0071958 (PMC3742455; doi:10.1371/journal.pone.0071958)

**Figure S1**

**
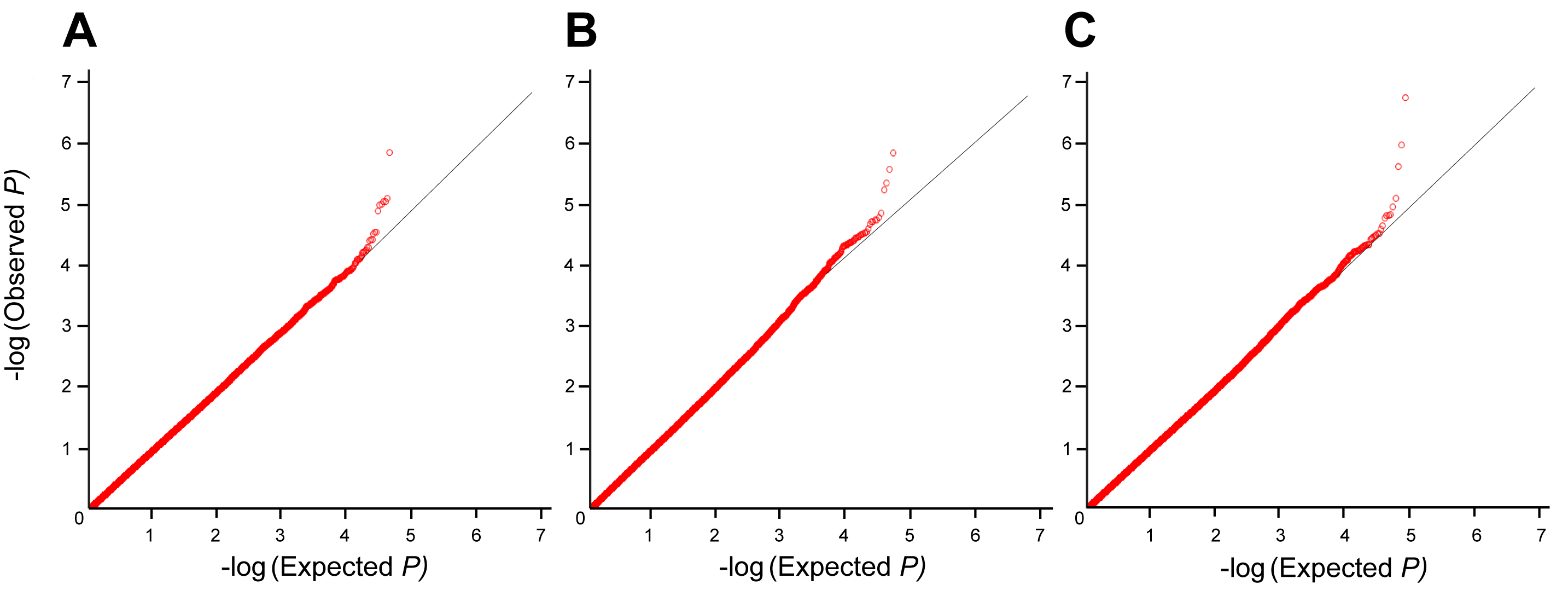
**

Supplement: Figure S1 — Q-Q plots of total IgE, specific IgE (D.p.), and specific IgE (D.f.). The observed P-value (y-axis) is compared with the expected P-value (x-axis, under null distribution) for (A) total IgE, (B) specific IgE (D.p.), and (C) specific IgE (D.f.). (DOC) [file pone.0071958.s001.doc]

**Figure S2**

**
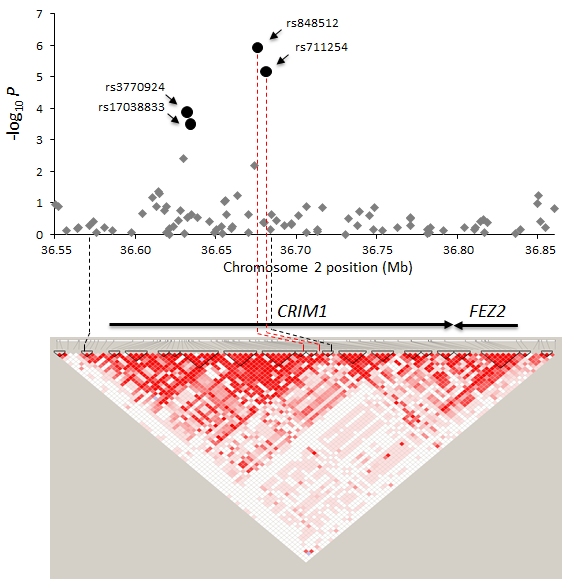
**

Supplement: Figure S2 — Regional association plot and LD of CRIM1 for the total IgE in asthmatics. GWAS associations (−log10 P) of SNPs across approximately 310 kb region around CRIM1 in the chromosome 2p21 are shown. Relatively strong associations are shown as large black circles; relatively less significances as small gray diamonds. (DOC) [file pone.0071958.s002.doc]

**Figure S3**

**
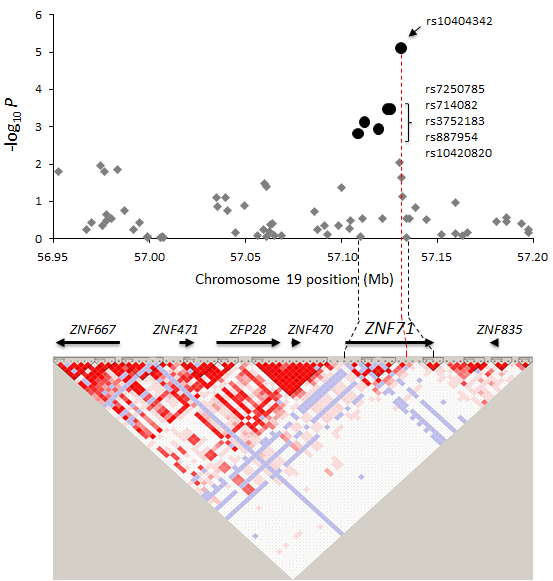
**

Supplement: Figure S3 — Regional association plot and LD of ZNF71 for the total IgE in asthmatics. GWAS associations (−log10 P) of SNPs across approximately 245 kb region around ZNF71 in the chromosome 19q13.4 are shown. Relatively strong associations are shown as large black circles; relatively less significances as small gray diamonds. (DOC) [file pone.0071958.s003.doc]

**Figure S4**

**
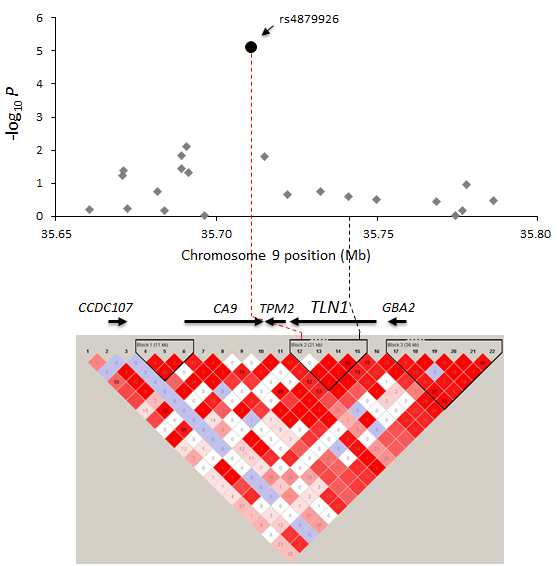
**

Supplement: Figure S4 — Regional association plot and LD of TLN1 for the total IgE in asthmatics. GWAS associations (−log10 P) of SNPs across approximately 126 kb region around TLN1 in the chromosome 9p13 are shown. Relatively strong associations are shown as large black circles; relatively less significances as small gray diamonds. (DOC) [file pone.0071958.s004.doc]

**Figure S5**


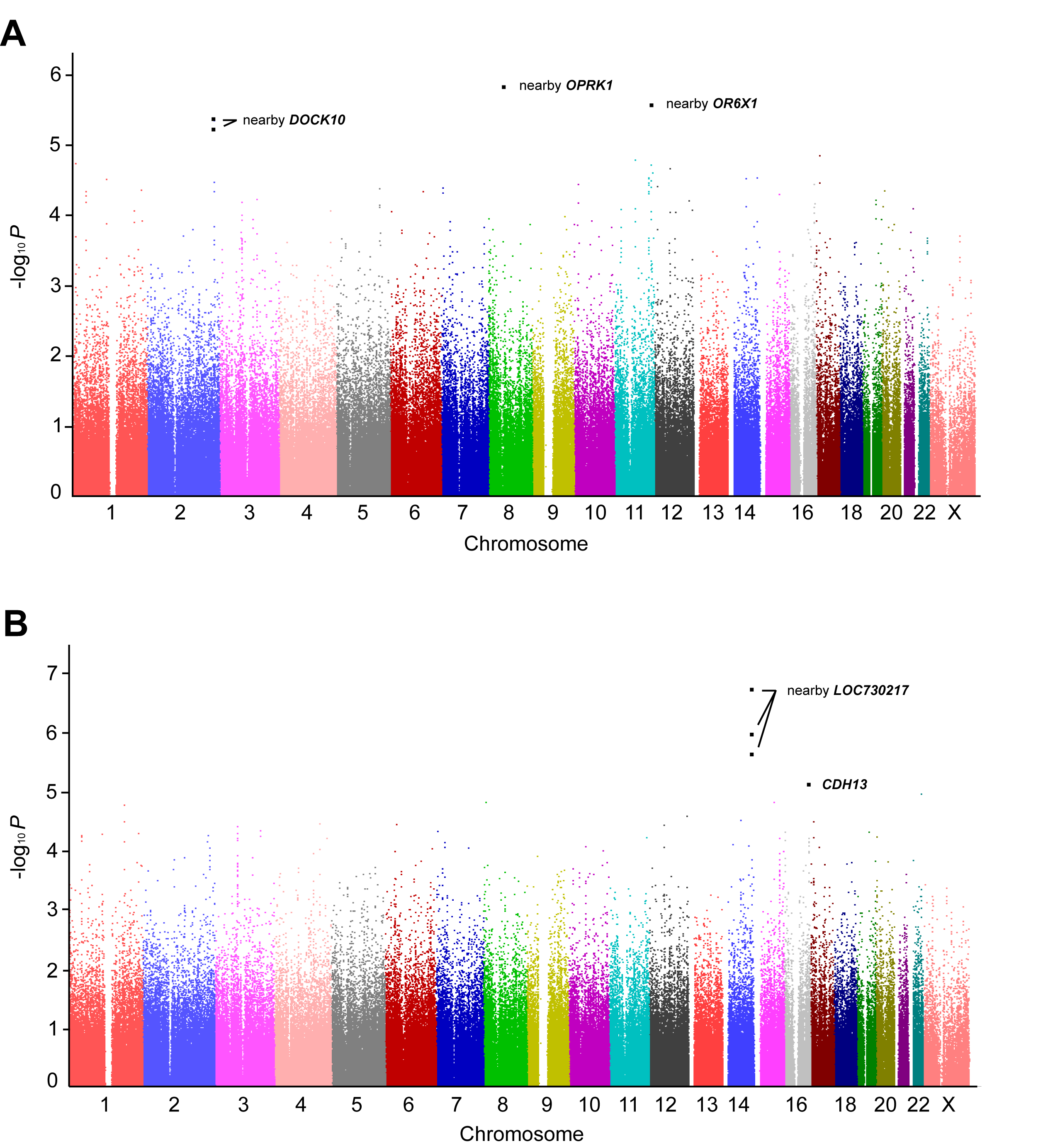

Supplement: Figure S5 — Manhattan plots of specific IgE against house dust mites (D.p. and D.f.). Associations for the specific IgE against (A) D.p. and (B) D.f. in asthma patients are obtained by correcting for age, gender, and smoking status as covariates. (DOC) [file pone.0071958.s005.doc]

**Figure S6**

**
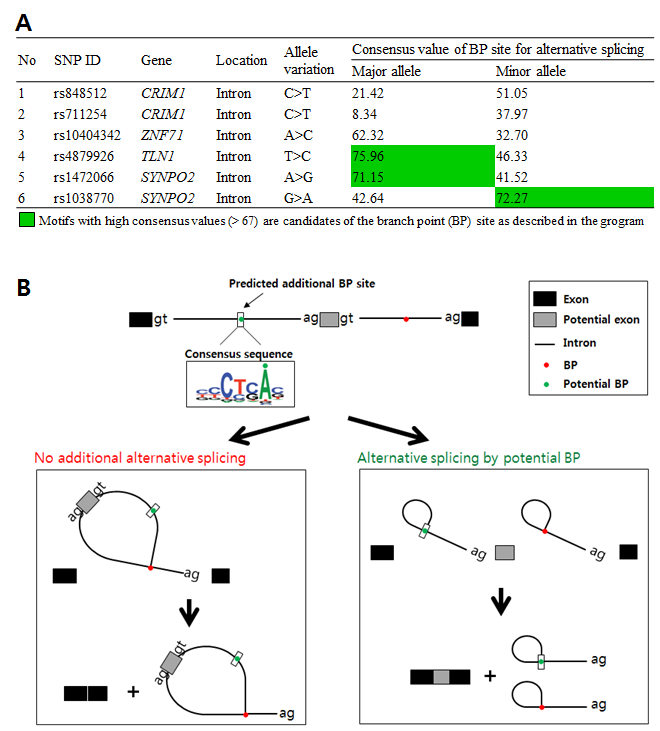
**

Supplement: Figure S6 — In silico analysis of top 6 SNPs in intron regions. (A) Potential branch point (BP) sites are predicted by the change between major allele and minor allele using the Human Splicing Finder (http://www.umd.be/HSF/). (B) A schematic plot of alternative splicing by the predicted additional BP site. The picture of consensus sequence is modified from Desmet et al. Nucleic Acids Research 37(9): e67, 2009. (DOC) [file pone.0071958.s006.doc]
