# Supplementary material for: A Genome-Wide Association Study of Total Serum and Mite-Specific IgEs in Asthma Patients
Source: PLoS One. 2013 Aug 13;8(8):e71958. doi: 10.1371/journal.pone.0071958 (PMC3742455; doi:10.1371/journal.pone.0071958)
Supplement: Table S1 — Classification of specific IgE. (DOC) [file pone.0071958.s007.doc]

**Table S1.** Classification of specific IgE

| Specific IgE class | ImmunoCAP specific IgE Units (kU/l) | Level of allergen specific IgE Ab |
| --- | --- | --- |
| 6 | >100 | Very high |
| 5 | 50-100 | Very high |
| 4 | 17.5-50 | Very high |
| 3 | 3.5-17.5 | High |
| 2 | 0.7-3.5 | Moderate |
| 1 | 0.35-0.7 | Low |
| 0 | 0.35 | Absent or undetectable |

Ab, antibody.
