# Supplementary material for: A Genome-Wide Association Study of Total Serum and Mite-Specific IgEs in Asthma Patients
Source: PLoS One. 2013 Aug 13;8(8):e71958. doi: 10.1371/journal.pone.0071958 (PMC3742455; doi:10.1371/journal.pone.0071958)
Supplement: Table S2 — Top 100 SNPs associated with total IgE of asthmatics in the GWAS. (DOC) [file pone.0071958.s008.doc]

**Table S2.** Top 100 SNPs associated with total IgE of asthmatics in the GWAS

|  |  |  |  |  |  |  | Genotype** | | |  |
| --- | --- | --- | --- | --- | --- | --- | --- | --- | --- | --- |
| SNP ID | Chr | (Nearby) gene | Location | Variation | MAF | HWE* | M/M† | M/m† | m/m† | *P*-value |
| rs848512 | 2 | *CRIM1* | Intron | C>T | 0.078 | 0.283 | 744 (2.22) | 130 (1.93) | 3 (1.89) | 1.18E-06 |
| rs711254 | 2 | *CRIM1* | Intron | C>T | 0.083 | 0.373 | 735 (2.22) | 137 (1.97) | 4 (1.76) | 6.73E-06 |
| rs10404342 | 19 | *ZNF71* | Intron | A>C | 0.399 | 0.748 | 315 (2.05) | 425 (2.23) | 137 (2.33) | 7.60E-06 |
| rs4879926 | 9 | *TLN1* | Intron | T>C | 0.072 | 0.070 | 751 (2.22) | 125 (1.96) | 1 (2.30) | 7.74E-06 |
| rs1472066 | 4 | *SYNPO2* | Intron | A>G | 0.107 | 0.744 | 700 (2.23) | 166 (1.99) | 11 (1.81) | 8.36E-06 |
| rs1038770 | 4 | *SYNPO2* | Intron | G>A | 0.116 | 0.805 | 685 (2.23) | 181 (2.02) | 11 (1.60) | 8.66E-06 |
| rs12508843 | 4 | *SYNPO2* | Intron | G>T | 0.193 | 0.597 | 574 (2.24) | 268 (2.09) | 35 (1.82) | 1.10E-05 |
| rs5766564 | 22 | *C22orf9* | Intron | G>A | 0.339 | 0.073 | 371 (2.26) | 417 (2.16) | 89 (1.95) | 2.48E-05 |
| rs7667661 | 4 | *NOL14* | Intron | G>A | 0.078 | 0.869 | 745 (2.21) | 127 (2.02) | 5 (1.62) | 2.50E-05 |
| rs1866990 | 4 | *GABRB1* | Intron | G>A | 0.270 | 0.858 | 466 (2.27) | 348 (2.08) | 63 (2.06) | 2.68E-05 |
| rs6683160 | 1 | *RYR2* | Intron | A>C | 0.348 | 0.638 | 376 (2.26) | 391 (2.16) | 109 (1.97) | 3.31E-05 |
| rs315782 | 5 | *(LCP2)* | Intergenic | T>C | 0.097 | 0.102 | 711 (2.14) | 162 (2.36) | 4 (2.34) | 3.36E-05 |
| rs777734 | 2 | *(LOC391470)* | Intergenic | C>A | 0.106 | 0.759 | 700 (2.22) | 168 (2.04) | 9 (1.61) | 4.39E-05 |
| rs1152937 | 12 | *CPM* | Intron | C>T | 0.145 | 0.337 | 637 (2.23) | 225 (2.07) | 15 (1.81) | 4.45E-05 |
| rs1990458 | 7 | *GCK* | Intron | G>A | 0.292 | 0.593 | 443 (2.10) | 356 (2.23) | 78 (2.38) | 5.24E-05 |
| rs17053323 | 4 | *(SPOCK3)* | Intergenic | A>G | 0.130 | 0.153 | 655 (2.23) | 207 (2.04) | 10 (1.96) | 5.37E-05 |
| rs10197865 | 2 | *GALNT14* | Intron | T>C | 0.380 | 0.003 | 358 (2.27) | 372 (2.14) | 147 (2.06) | 5.48E-05 |
| rs1168745 | 12 | *TMBIM4* | Intron | T>C | 0.186 | 0.608 | 579 (2.24) | 270 (2.08) | 28 (1.91) | 6.21E-05 |
| rs10911203 | 1 | *LAMC1* | Intron | G>A | 0.133 | 0.889 | 659 (2.23) | 203 (2.06) | 15 (1.87) | 6.53E-05 |
| rs395157 | 5 | *OSMR* | Intron | G>A | 0.265 | 0.558 | 471 (2.24) | 348 (2.14) | 58 (1.93) | 6.94E-05 |
| rs3757840 | 7 | *(GCK)* | Intergenic | A>C | 0.405 | 0.644 | 314 (2.07) | 416 (2.21) | 147 (2.32) | 7.05E-05 |
| rs9549433 | 13 | *(LOC729095)* | Intergenic | C>A | 0.251 | 0.271 | 484 (2.12) | 341 (2.24) | 49 (2.38) | 7.40E-05 |
| rs7608731 | 2 | *GALNT14* | Intron | C>T | 0.379 | 0.004 | 358 (2.27) | 373 (2.14) | 146 (2.07) | 8.15E-05 |
| rs5766584 | 22 | *(C22orf9)* | Intergenic | G>T | 0.460 | 0.046 | 241 (2.04) | 465 (2.23) | 171 (2.25) | 9.05E-05 |
| rs11090735 | 22 | *C22orf9* | Intron | T>C | 0.324 | 0.041 | 387 (2.25) | 411 (2.16) | 79 (1.95) | 9.64E-05 |
| rs9308914 | 2 | *GALNT14* | Intron | A>C | 0.388 | 0.010 | 347 (2.27) | 380 (2.15) | 150 (2.05) | 0.00010 |
| rs9955902 | 18 | *(MC5R)* | Intergenic | T>G | 0.396 | 0.065 | 307 (2.11) | 445 (2.17) | 124 (2.38) | 0.00010 |
| rs4610128 | 20 | *(LOC441940)* | Intergenic | G>A | 0.115 | 0.402 | 685 (2.22) | 183 (2.04) | 9 (1.75) | 0.00011 |
| rs996384 | 20 | *(LOC441940)* | Intergenic | T>G | 0.115 | 0.402 | 685 (2.22) | 183 (2.04) | 9 (1.75) | 0.00011 |
| rs12136061 | 1 | *(LOC730175)* | Intergenic | C>T | 0.151 | 0.121 | 627 (2.12) | 236 (2.33) | 14 (2.28) | 0.00011 |
| rs17479287 | 1 | *(LAMC2)* | Intergenic | G>A | 0.078 | 0.198 | 749 (2.21) | 120 (2.01) | 8 (1.76) | 0.00011 |
| rs10836063 | 11 | *(HIPK3)* | Intergenic | C>T | 0.210 | 0.970 | 547 (2.11) | 291 (2.30) | 39 (2.26) | 0.00011 |
| rs1862968 | 2 | *GALNT14* | Intron | A>G | 0.383 | 0.002 | 355 (2.27) | 370 (2.14) | 150 (2.07) | 0.00011 |
| rs7170834 | 15 | *(LOC390641)* | Intergenic | G>T | 0.179 | 0.113 | 598 (2.22) | 244 (2.12) | 35 (1.93) | 0.00012 |
| rs6875477 | 5 | *(LOC728878)* | Intergenic | A>G | 0.214 | 0.952 | 541 (2.11) | 296 (2.29) | 40 (2.32) | 0.00012 |
| rs2074920 | 19 | *SBNO2* | Coding | C>T | 0.320 | 0.790 | 404 (2.25) | 385 (2.13) | 88 (2.07) | 0.00012 |
| rs12536378 | 7 | *NULL* | Intron | T>C | 0.304 | 0.665 | 427 (2.23) | 366 (2.19) | 84 (1.87) | 0.00013 |
| rs7211689 | 17 | *(FLJ45831)* | Intergenic | A>C | 0.404 | 0.921 | 312 (2.08) | 421 (2.21) | 144 (2.30) | 0.00013 |
| rs1698157 | 11 | *(LOC283143)* | Intergenic | C>A | 0.477 | 0.072 | 253 (2.06) | 411 (2.21) | 213 (2.26) | 0.00013 |
| rs7298821 | 12 | *CACNA1C* | Intron | C>T | 0.302 | 0.084 | 417 (2.27) | 391 (2.11) | 69 (2.04) | 0.00013 |
| rs3770924 | 2 | *CRIM1* | Intron | C>T | 0.076 | 0.325 | 747 (2.21) | 127 (2.00) | 3 (1.89) | 0.00013 |
| rs10911180 | 1 | *(KRT18P28)* | Intergenic | C>A | 0.125 | 0.836 | 671 (2.22) | 193 (2.05) | 13 (1.87) | 0.00014 |
| rs2113097 | 5 | *SLC45A2* | Intron | G>A | 0.227 | 0.722 | 526 (2.11) | 304 (2.27) | 47 (2.35) | 0.00014 |
| rs2287949 | 5 | *SLC45A2* | Coding | C>T | 0.227 | 0.722 | 526 (2.11) | 304 (2.27) | 47 (2.35) | 0.00014 |
| rs7967521 | 12 | *SCARB1* | Intron | T>C | 0.321 | 0.715 | 402 (2.11) | 387 (2.22) | 88 (2.33) | 0.00014 |
| rs10495085 | 1 | *(SPATA17)* | Intergenic | C>T | 0.060 | 0.904 | 774 (2.15) | 100 (2.36) | 3 (3.22) | 0.00014 |
| rs7494167 | 14 | *(DIO3OS)* | Intergenic | G>A | 0.194 | 0.643 | 567 (2.24) | 279 (2.10) | 31 (1.90) | 0.00015 |
| rs2072280 | 19 | *SBNO2* | Coding | G>A | 0.319 | 0.842 | 404 (2.25) | 383 (2.13) | 88 (2.07) | 0.00015 |
| rs1010872 | 5 | *SLC45A2* | Intron | C>A | 0.226 | 0.622 | 526 (2.11) | 300 (2.27) | 47 (2.35) | 0.00015 |
| rs11159411 | 14 | *(LOC401767)* | Intergenic | C>T | 0.223 | 0.935 | 530 (2.12) | 303 (2.25) | 44 (2.41) | 0.00015 |
| rs994565 | 6 | *(EDN1)* | Intergenic | C>T | 0.457 | 0.648 | 255 (2.26) | 442 (2.18) | 180 (2.06) | 0.00015 |
| rs4689941 | 4 | *(MSX1)* | Intergenic | C>A | 0.327 | 0.794 | 395 (2.10) | 389 (2.23) | 92 (2.34) | 0.00016 |
| rs7787692 | 7 | *(FZD1)* | Intergenic | C>A | 0.124 | 0.429 | 670 (2.14) | 196 (2.32) | 11 (2.48) | 0.00016 |
| rs1531160 | 3 | *(LOC728516)* | Intergenic | T>C | 0.187 | 0.160 | 586 (2.12) | 254 (2.29) | 37 (2.32) | 0.00016 |
| rs1046279 | 6 | *PI16* | 3'UTR | T>C | 0.489 | 0.799 | 231 (2.30) | 434 (2.16) | 211 (2.09) | 0.00016 |
| rs7694687 | 4 | *(HTT)* | Intergenic | C>T | 0.086 | 0.283 | 730 (2.21) | 143 (2.05) | 4 (1.45) | 0.00017 |

**Table S2.** Continued

| rs7865631 | 9 | *(CYLC2)* | Intergenic | A>G | 0.060 | 0.904 | 774 (2.21) | 100 (1.97) | 3 (2.09) | 0.00017 |
| --- | --- | --- | --- | --- | --- | --- | --- | --- | --- | --- |
| rs7141155 | 14 | *SIPA1L1* | Intron | G>A | 0.190 | 0.630 | 577 (2.24) | 266 (2.08) | 34 (1.99) | 0.00018 |
| rs8042861 | 15 | *IQGAP1* | Intron | T>G | 0.142 | 0.455 | 642 (2.23) | 219 (2.04) | 15 (2.06) | 0.00018 |
| rs1956967 | 14 | *SFTPH* | Intron | C>A | 0.074 | 0.283 | 754 (2.22) | 116 (1.95) | 7 (2.18) | 0.00018 |
| rs1914748 | 2 | *FHL2* | Intron | T>C | 0.404 | 0.066 | 325 (2.27) | 396 (2.16) | 156 (2.04) | 0.00019 |
| rs11140441 | 9 | *(SLC28A3)* | Intergenic | A>G | 0.117 | 0.509 | 686 (2.21) | 177 (2.12) | 14 (1.55) | 0.00020 |
| rs6540844 | 1 | *(LOC643454)* | Intergenic | T>C | 0.461 | 0.100 | 243 (2.09) | 460 (2.18) | 174 (2.29) | 0.00020 |
| rs3924902 | 12 | *(TMEM132C)* | Intergenic | T>C | 0.318 | 0.704 | 406 (2.09) | 385 (2.23) | 86 (2.34) | 0.00020 |
| rs17436172 | 1 | *IGSF21* | Intron | A>G | 0.441 | 0.157 | 264 (2.26) | 453 (2.17) | 160 (2.06) | 0.00020 |
| rs12197082 | 6 | *(FAM50B)* | Intergenic | G>A | 0.103 | 0.393 | 703 (2.15) | 167 (2.31) | 7 (2.58) | 0.00021 |
| rs1566652 | 16 | *SLC6A2* | Intron | G>T | 0.334 | 0.408 | 395 (2.09) | 379 (2.22) | 103 (2.36) | 0.00021 |
| rs10858366 | 9 | *(OLFM1)* | Intergenic | A>C | 0.105 | 0.930 | 702 (2.21) | 165 (2.04) | 10 (2.00) | 0.00021 |
| rs1127065 | 7 | *CAMK2B* | Coding | G>A | 0.310 | 0.796 | 419 (2.10) | 372 (2.24) | 86 (2.33) | 0.00022 |
| rs12263637 | 10 | *(ZWINT)* | Intergenic | A>G | 0.137 | 0.194 | 648 (2.22) | 217 (2.08) | 12 (1.80) | 0.00022 |
| rs2358866 | 10 | *PLXDC2* | Intron | G>A | 0.432 | 0.078 | 270 (2.31) | 456 (2.13) | 151 (2.09) | 0.00022 |
| rs2884567 | 10 | *PLXDC2* | Intron | G>A | 0.432 | 0.078 | 270 (2.31) | 456 (2.13) | 151 (2.09) | 0.00022 |
| rs1698193 | 11 | *(LOC283143)* | Intergenic | G>T | 0.417 | 0.252 | 306 (2.08) | 410 (2.23) | 161 (2.25) | 0.00022 |
| rs1040303 | 14 | *(TRDV2)* | Intergenic | G>A | 0.154 | 0.215 | 623 (2.14) | 238 (2.27) | 16 (2.52) | 0.00022 |
| rs714795 | 14 | *(TRDV2)* | Intergenic | G>A | 0.154 | 0.215 | 623 (2.14) | 238 (2.27) | 16 (2.52) | 0.00022 |
| rs4629410 | 4 | *GRID2* | Intron | A>G | 0.071 | 0.221 | 755 (2.21) | 120 (1.99) | 2 (1.57) | 0.00023 |
| rs12565776 | 1 | *(LAMC1)* | Intergenic | A>G | 0.116 | 0.964 | 685 (2.22) | 180 (2.06) | 12 (1.81) | 0.00023 |
| rs6543594 | 2 | *GALNT14* | Intron | C>A | 0.376 | 0.004 | 361 (2.26) | 372 (2.14) | 144 (2.07) | 0.00023 |
| rs571877 | 17 | *(C17orf76)* | Intergenic | T>C | 0.493 | 0.402 | 232 (2.08) | 426 (2.18) | 219 (2.29) | 0.00023 |
| rs2449215 | 8 | *CSMD1* | Intron | C>T | 0.386 | 0.633 | 334 (2.25) | 409 (2.16) | 134 (2.05) | 0.00023 |
| rs11075997 | 16 | *FTO* | Intron | C>T | 0.161 | 0.770 | 618 (2.23) | 235 (2.08) | 24 (1.95) | 0.00024 |
| rs2120663 | 18 | *OSBPL1A* | Intron | C>T | 0.275 | 0.554 | 464 (2.12) | 343 (2.23) | 70 (2.34) | 0.00024 |
| rs1489259 | 1 | *(LOC730175)* | Intergenic | A>G | 0.168 | 0.261 | 603 (2.11) | 254 (2.34) | 20 (2.17) | 0.00024 |
| rs2072268 | 17 | *(ARSG)* | Intergenic | T>C | 0.326 | 0.764 | 400 (2.28) | 381 (2.10) | 95 (2.10) | 0.00024 |
| rs10911167 | 1 | *(KRT18P28)* | Intergenic | T>C | 0.124 | 0.865 | 672 (2.22) | 192 (2.06) | 13 (1.87) | 0.00024 |
| rs4518993 | 10 | *PLXDC2* | Intron | G>A | 0.477 | 0.328 | 233 (2.30) | 452 (2.16) | 192 (2.07) | 0.00025 |
| rs1338078 | 1 | *(LOC643329)* | Intergenic | G>T | 0.432 | 0.150 | 292 (2.06) | 407 (2.23) | 173 (2.25) | 0.00025 |
| rs471728 | 11 | *(LOC729717)* | Intergenic | G>A | 0.475 | 0.914 | 241 (2.27) | 439 (2.20) | 197 (2.03) | 0.00025 |
| rs2322732 | 11 | *(PRR10)* | Intergenic | G>A | 0.121 | 0.983 | 677 (2.22) | 187 (2.07) | 13 (1.87) | 0.00025 |
| rs7299020 | 12 | *(LRIG3)* | Intergenic | A>G | 0.278 | 0.627 | 454 (2.26) | 358 (2.12) | 65 (1.96) | 0.00026 |
| rs11177048 | 12 | *LOC341333* | Intron | G>A | 0.081 | 0.212 | 738 (2.15) | 136 (2.36) | 3 (2.43) | 0.00026 |
| rs780827 | 10 | *CUBN* | Intron | C>T | 0.417 | 0.754 | 300 (2.12) | 422 (2.17) | 155 (2.34) | 0.00026 |
| rs11011827 | 10 | *PLXDC2* | Intron | C>A | 0.481 | 0.260 | 228 (2.31) | 454 (2.16) | 194 (2.07) | 0.00026 |
| rs8002916 | 13 | *(POSTN)* | Intergenic | A>G | 0.429 | 0.000 | 328 (2.28) | 340 (2.13) | 204 (2.10) | 0.00026 |
| rs480836 | 1 | *DAB1* | Intron | C>T | 0.273 | 0.434 | 468 (2.23) | 339 (2.16) | 70 (1.94) | 0.00027 |
| rs9307398 | 4 | *(ARSJ)* | Intergenic | A>G | 0.456 | 0.449 | 265 (2.29) | 424 (2.15) | 188 (2.08) | 0.00027 |
| rs9972327 | 15 | *IDH2* | Intron | C>A | 0.245 | 0.318 | 495 (2.24) | 335 (2.13) | 47 (1.95) | 0.00027 |
| rs11625806 | 14 | *(LOC729042)* | Intergenic | G>A | 0.079 | 0.506 | 743 (2.21) | 130 (2.03) | 4 (1.66) | 0.00027 |
| rs2350064 | 4 | *(EPHA5)* | Intergenic | C>T | 0.161 | 0.836 | 616 (2.22) | 239 (2.09) | 22 (1.96) | 0.00027 |
| rs17288177 | 3 | *(GRM7)* | Intergenic | T>C | 0.358 | 0.602 | 357 (2.25) | 410 (2.16) | 109 (2.03) | 0.00027 |

Association analyses were adjusted by age, sex, and smoking status as covariates.

**P*-value of Hardy-Weinberg equilibrium (HWE).

**Genotype represents number of subjects (mean of log[Total IgE (IU/ml)]).

†M/M, M/m, and m/m indicate homozygote for common allele, heterozygote, and homozygote for rare allele, respectively.

Chr, chromosome; MAF, minor allele frequency.
