# Supplementary material for: A Genome-Wide Association Study of Total Serum and Mite-Specific IgEs in Asthma Patients
Source: PLoS One. 2013 Aug 13;8(8):e71958. doi: 10.1371/journal.pone.0071958 (PMC3742455; doi:10.1371/journal.pone.0071958)
Supplement: Table S3 — Top 100 SNPs associated with specific IgE for D.p. in the GWAS. (DOC) [file pone.0071958.s009.doc]

**Table S3.** Top 100 SNPs associated with specific IgE for D.p. in the GWAS

|  |  |  |  |  |  | MAF | |  |  |
| --- | --- | --- | --- | --- | --- | --- | --- | --- | --- |
|  |  |  |  |  |  | Specific IgE (D.p.) | |  |  |
| SNP ID | Chr | (Nearby) gene | Location | Variation | HWE* | Positive | Negative | OR (95% CI) | *P*-value |
| rs1425902 | 8 | *(OPRK1)* | Intergenic | A>G | 0.069 | 0.373 | 0.262 | 2.14 (1.56-2.94) | 1.44E-06 |
| rs17744026 | 11 | *(OR6X1)* | Intergenic | T>G | 0.866 | 0.035 | 0.095 | 0.25 (0.13-0.48) | 2.66E-06 |
| rs1843834 | 2 | *(DOCK10)* | Intergenic | G>A | 0.858 | 0.286 | 0.183 | 2.14 (1.54-2.96) | 4.38E-06 |
| rs10197382 | 2 | *(DOCK10)* | Intergenic | C>T | 0.063 | 0.332 | 0.229 | 2.09 (1.52-2.89) | 5.72E-06 |
| rs12952334 | 17 | *MYH2* | Intron | G>A | 0.478 | 0.182 | 0.111 | 2.38 (1.61-3.51) | 1.40E-05 |
| rs7105655 | 11 | *(FGF4)* | Intergenic | C>A | 0.340 | 0.159 | 0.255 | 0.47 (0.33-0.67) | 1.61E-05 |
| rs11219307 | 11 | *(OR6M1)* | Intergenic | G>T | 0.550 | 0.113 | 0.203 | 0.43 (0.29-0.65) | 1.90E-05 |
| rs2155362 | 11 | *(OR6M1)* | Intergenic | G>A | 0.550 | 0.113 | 0.203 | 0.43 (0.29-0.65) | 1.90E-05 |
| rs1566869 | 12 | *(ANKRD33)* | Intergenic | A>G | 0.315 | 0.428 | 0.314 | 1.83 (1.38-2.43) | 2.12E-05 |
| rs10791006 | 11 | *BARX2* | Intron | T>C | 0.192 | 0.575 | 0.474 | 1.78 (1.35-2.33) | 2.45E-05 |
| rs10142119 | 14 | *(LOC730217)* | Intergenic | G>A | 0.527 | 0.344 | 0.471 | 0.55 (0.42-0.73) | 2.86E-05 |
| rs2155633 | 11 | *(CADM1)* | Intergenic | G>A | 0.094 | 0.535 | 0.427 | 1.76 (1.35-2.31) | 2.91E-05 |
| rs2846895 | 11 | *(CADM1)* | Intergenic | G>A | 0.518 | 0.477 | 0.372 | 1.80 (1.36-2.37) | 2.93E-05 |
| rs2273623 | 14 | *(PPM1A)* | Intergenic | A>G | 0.614 | 0.145 | 0.076 | 2.59 (1.66-4.02) | 2.98E-05 |
| rs12273131 | 11 | *(PMP22CD)* | Intergenic | C>T | 0.708 | 0.118 | 0.210 | 0.45 (0.30-0.67) | 3.14E-05 |
| rs1565073 | 2 | *DOCK10* | Intron | G>T | 0.007 | 0.298 | 0.208 | 2.05 (1.46-2.88) | 3.31E-05 |
| rs2108606 | 17 | *MYH4* | Intron | T>C | 0.346 | 0.176 | 0.109 | 2.29 (1.55-3.39) | 3.37E-05 |
| rs2613468 | 11 | *(CADM1)* | Intergenic | G>A | 0.024 | 0.532 | 0.424 | 1.74 (1.34-2.28) | 3.39E-05 |
| rs7900357 | 10 | *FRMD4A* | Intron | G>A | 0.859 | 0.225 | 0.327 | 0.53 (0.38-0.72) | 3.58E-05 |
| rs6563898 | 16 | *CDH13* | Intron | G>A | 0.211 | 0.384 | 0.480 | 0.55 (0.41-0.74) | 3.60E-05 |
| rs1785016 | 11 | *(LOC283143)* | Intergenic | G>A | 0.256 | 0.434 | 0.343 | 1.79 (1.35-2.36) | 3.64E-05 |
| rs12283877 | 11 | *(PMP22CD)* | Intergenic | C>T | 0.694 | 0.116 | 0.201 | 0.45 (0.30-0.67) | 3.88E-05 |
| rs987651 | 5 | *STK32A* | Intron | C>T | 0.186 | 0.572 | 0.460 | 1.79 (1.35-2.38) | 4.14E-05 |
| rs446924 | 1 | *ABCB10* | Intron | C>T | 0.013 | 0.055 | 0.126 | 0.37 (0.22-0.62) | 4.32E-05 |
| rs4813955 | 20 | *(hCG_2045828)* | Intergenic | A>C | 0.196 | 0.341 | 0.253 | 1.85 (1.38-2.50) | 4.44E-05 |
| rs1698157 | 11 | *(LOC283143)* | Intergenic | C>A | 0.028 | 0.549 | 0.447 | 1.73 (1.32-2.26) | 4.49E-05 |
| rs6717059 | 2 | *DOCK10* | Intron | T>C | 0.011 | 0.269 | 0.184 | 2.08 (1.46-2.95) | 4.54E-05 |
| rs3799852 | 6 | *FIG4* | Intron | G>A | 0.923 | 0.358 | 0.252 | 1.85 (1.37-2.49) | 4.55E-05 |
| rs12024772 | 1 | *HIVEP3* | Intron | G>A | 0.960 | 0.410 | 0.514 | 0.56 (0.42-0.74) | 4.55E-05 |
| rs2155631 | 11 | *(LOC283143)* | Intergenic | T>G | 0.115 | 0.561 | 0.461 | 1.73 (1.32-2.27) | 4.89E-05 |
| rs4776381 | 15 | *(LBXCOR1)* | Intergenic | G>A | 0.120 | 0.179 | 0.089 | 2.23 (1.52-3.28) | 4.92E-05 |
| rs2993120 | 1 | *HIVEP3* | Intron | A>C | 0.880 | 0.419 | 0.520 | 0.56 (0.42-0.75) | 5.17E-05 |
| rs3736388 | 3 | *(KALRN)* | Intergenic | T>C | 0.181 | 0.237 | 0.374 | 0.55 (0.41-0.74) | 5.87E-05 |
| rs3810385 | 19 | *(DYRK1B)* | Intergenic | A>G | 0.217 | 0.023 | 0.078 | 0.24 (0.11-0.53) | 6.00E-05 |
| rs2195003 | 12 | *(MED13L)* | Intergenic | A>G | 0.218 | 0.283 | 0.402 | 0.55 (0.41-0.74) | 6.11E-05 |
| rs11210537 | 1 | *HIVEP3* | Intron | A>G | 0.910 | 0.422 | 0.520 | 0.57 (0.43-0.75) | 6.39E-05 |
| rs12630469 | 3 | *(CNTN3)* | Intergenic | A>G | 0.978 | 0.266 | 0.389 | 0.55 (0.40-0.74) | 6.41E-05 |
| rs7905868 | 10 | *(FAM107B)* | Intergenic | T>C | 0.255 | 0.338 | 0.240 | 1.83 (1.36-2.46) | 6.64E-05 |
| rs2247470 | 16 | *NECAB2* | Intron | A>G | 0.690 | 0.079 | 0.144 | 0.40 (0.25-0.65) | 6.73E-05 |
| rs3810384 | 19 | *(DYRK1B)* | Intergenic | G>A | 0.227 | 0.023 | 0.077 | 0.24 (0.11-0.54) | 6.86E-05 |
| rs13170855 | 5 | *STK32A* | Intron | T>C | 0.601 | 0.529 | 0.418 | 1.74 (1.32-2.30) | 7.10E-05 |
| rs1887104 | 14 | *PPM1A* | Intron | A>C | 0.922 | 0.116 | 0.059 | 2.71 (1.67-4.42) | 7.34E-05 |
| rs2423011 | 20 | *(RASSF2)* | Intergenic | A>G | 0.319 | 0.413 | 0.515 | 0.58 (0.45-0.77) | 7.38E-05 |
| rs1549881 | 5 | *STK32A* | Intron | G>A | 0.411 | 0.500 | 0.384 | 1.74 (1.32-2.30) | 7.60E-05 |
| rs763727 | 16 | *CDH13* | Intron | A>G | 0.792 | 0.564 | 0.474 | 1.75 (1.32-2.32) | 7.61E-05 |
| rs11263535 | 11 | *(FGF19)* | Intergenic | A>G | 0.668 | 0.214 | 0.131 | 2.07 (1.44-2.97) | 8.04E-05 |
| rs12283302 | 11 | *SLC6A5* | Intron | T>G | 0.959 | 0.237 | 0.319 | 0.54 (0.39-0.74) | 8.15E-05 |
| rs10773370 | 12 | *(LOC121296)* | Intergenic | C>T | 0.577 | 0.590 | 0.468 | 1.74 (1.31-2.30) | 8.26E-05 |
| rs7515178 | 1 | *RBBP5* | Intron | G>A | 0.494 | 0.104 | 0.054 | 2.85 (1.70-4.80) | 8.51E-05 |
| rs9502039 | 6 | *(C6orf145)* | Intergenic | C>T | 0.165 | 0.373 | 0.272 | 1.76 (1.32-2.33) | 8.71E-05 |
| rs1939915 | 11 | *(PMP22CD)* | Intergenic | T>C | 0.650 | 0.171 | 0.265 | 0.51 (0.36-0.72) | 8.80E-05 |
| rs2155363 | 11 | *(PMP22CD)* | Intergenic | T>C | 0.650 | 0.171 | 0.265 | 0.51 (0.36-0.72) | 8.80E-05 |
| rs609704 | 12 | *(KRT71)* | Intergenic | T>C | 0.805 | 0.552 | 0.453 | 1.73 (1.31-2.28) | 8.96E-05 |
| rs4294640 | 12 | *PTPRR* | Intron | G>T | 0.824 | 0.439 | 0.315 | 1.75 (1.32-2.32) | 9.00E-05 |
| rs7194029 | 16 | *CDH13* | Intron | C>T | 0.500 | 0.549 | 0.459 | 1.74 (1.31-2.32) | 9.19E-05 |
| rs9828201 | 3 | *(LOC728135)* | Intergenic | T>C | 0.790 | 0.286 | 0.424 | 0.56 (0.42-0.76) | 9.86E-05 |

**Table S3.** Continued

| rs7863292 | 9 | *(LOC644723)* | Intergenic | G>A | 0.707 | 0.017 | 0.071 | 0.23 (0.10-0.56) | 0.00010 |
| --- | --- | --- | --- | --- | --- | --- | --- | --- | --- |
| rs1017078 | 19 | *(DYRK1B)* | Intergenic | G>A | 0.188 | 0.026 | 0.079 | 0.26 (0.12-0.57) | 0.00011 |
| rs2957086 | 8 | *DLGAP2* | Intron | A>G | 0.648 | 0.338 | 0.456 | 0.57 (0.43-0.77) | 0.00011 |
| rs1398748 | 3 | *CD96* | Intron | T>C | 0.249 | 0.416 | 0.521 | 0.59 (0.45-0.78) | 0.00011 |
| rs1275054 | 11 | *(ZNF202)* | Intergenic | G>A | 0.391 | 0.038 | 0.090 | 0.32 (0.17-0.61) | 0.00011 |
| rs1029709 | 19 | *(ZFP28)* | Intergenic | C>T | 0.173 | 0.185 | 0.277 | 0.52 (0.37-0.73) | 0.00011 |
| rs7629942 | 3 | *(CNTN3)* | Intergenic | G>A | 0.766 | 0.272 | 0.391 | 0.56 (0.41-0.76) | 0.00012 |
| rs4420916 | 3 | *(CNTN3)* | Intergenic | G>A | 0.766 | 0.272 | 0.391 | 0.56 (0.41-0.76) | 0.00012 |
| rs6795835 | 3 | *(LOC728135)* | Intergenic | C>T | 0.766 | 0.272 | 0.391 | 0.56 (0.41-0.76) | 0.00012 |
| rs16854967 | 1 | *DISC1* | Intron | A>G | 0.403 | 0.038 | 0.118 | 0.33 (0.18-0.62) | 0.00012 |
| rs3098360 | 10 | *(ZWINT)* | Intergenic | T>C | 0.326 | 0.098 | 0.046 | 2.87 (1.69-4.86) | 0.00012 |
| rs2244811 | 17 | *(NXN)* | Intergenic | A>G | 0.198 | 0.153 | 0.082 | 2.39 (1.54-3.72) | 0.00012 |
| rs2286992 | 7 | *CPVL* | Intron | C>T | 0.186 | 0.257 | 0.379 | 0.56 (0.41-0.76) | 0.00012 |
| rs4980684 | 11 | *(FGF19)* | Intergenic | C>T | 0.994 | 0.257 | 0.169 | 1.92 (1.38-2.68) | 0.00012 |
| rs1456988 | 14 | *(LOC730217)* | Intergenic | T>G | 0.692 | 0.572 | 0.463 | 1.70 (1.29-2.24) | 0.00012 |
| rs6593920 | 1 | *CNTN2* | Intron | T>G | 0.543 | 0.147 | 0.088 | 2.30 (1.51-3.51) | 0.00012 |
| rs1035569 | 16 | *CDH13* | Intron | G>A | 0.496 | 0.546 | 0.458 | 1.73 (1.30-2.29) | 0.00013 |
| rs723919 | 16 | *CDH13* | Intron | A>G | 0.628 | 0.566 | 0.477 | 1.72 (1.30-2.29) | 0.00013 |
| rs1343817 | 1 | *CD53* | Intron | A>C | 0.933 | 0.556 | 0.438 | 1.70 (1.29-2.23) | 0.00013 |
| rs2255522 | 8 | *(KHDRBS3)* | Intergenic | C>T | 0.355 | 0.419 | 0.516 | 1.70 (1.29-2.24) | 0.00013 |
| rs4881399 | 10 | *(DIP2C)* | Intergenic | G>A | 0.841 | 0.049 | 0.119 | 0.36 (0.20-0.64) | 0.00013 |
| rs4812609 | 20 | *PTPRT* | Intron | G>A | 0.737 | 0.419 | 0.524 | 1.71 (1.29-2.26) | 0.00013 |
| rs4750112 | 10 | *(ECHDC3)* | Intergenic | G>A | 0.211 | 0.220 | 0.307 | 0.54 (0.39-0.75) | 0.00014 |
| rs10498606 | 14 | *(LOC283585)* | Intergenic | C>A | 0.598 | 0.546 | 0.421 | 1.69 (1.28-2.22) | 0.00014 |
| rs6731785 | 2 | *(CUL3)* | Intergenic | G>A | 0.712 | 0.263 | 0.183 | 1.89 (1.36-2.62) | 0.00014 |
| rs6948622 | 7 | *(LOC728466)* | Intergenic | G>A | 0.493 | 0.301 | 0.194 | 1.83 (1.34-2.51) | 0.00015 |
| rs17663304 | 10 | *(BUB3)* | Intergenic | T>C | 0.299 | 0.040 | 0.099 | 0.32 (0.17-0.61) | 0.00015 |
| rs10902907 | 10 | *(BUB3)* | Intergenic | C>T | 0.299 | 0.040 | 0.099 | 0.32 (0.17-0.61) | 0.00015 |
| rs4813479 | 20 | *(CD93)* | Intergenic | C>T | 0.684 | 0.292 | 0.180 | 1.86 (1.35-2.57) | 0.00015 |
| rs10741848 | 11 | *SLC6A5* | Intron | C>T | 0.653 | 0.240 | 0.327 | 0.56 (0.41-0.76) | 0.00015 |
| rs2173318 | 3 | *CD96* | Intron | T>C | 0.220 | 0.416 | 0.520 | 0.60 (0.46-0.78) | 0.00015 |
| rs12929441 | 16 | *(LOC643358)* | Intergenic | T>C | 0.805 | 0.191 | 0.108 | 2.10 (1.43-3.09) | 0.00016 |
| rs784932 | 9 | *(RGS3)* | Intergenic | G>A | 0.861 | 0.176 | 0.254 | 0.52 (0.36-0.74) | 0.00016 |
| rs11203582 | 8 | *SGCZ* | Intron | G>A | 0.698 | 0.286 | 0.383 | 0.57 (0.42-0.77) | 0.00016 |
| rs2053273 | 2 | *(RPRM)* | Intergenic | G>A | 0.554 | 0.390 | 0.512 | 0.59 (0.45-0.78) | 0.00016 |
| rs2815124 | 6 | *KCNK5* | Intron | C>T | 0.631 | 0.098 | 0.050 | 2.83 (1.66-4.83) | 0.00016 |
| rs12670165 | 7 | *CPVL* | Intron | C>T | 0.226 | 0.257 | 0.377 | 0.57 (0.42-0.77) | 0.00016 |
| rs1569907 | 20 | *C20orf77* | Intron | A>G | 0.254 | 0.188 | 0.106 | 2.14 (1.45-3.16) | 0.00016 |
| rs2268251 | 21 | *ITSN1* | Intron | G>T | 0.283 | 0.289 | 0.400 | 0.58 (0.43-0.77) | 0.00017 |
| rs4677048 | 3 | *(PROK2)* | Intergenic | A>G | 0.818 | 0.237 | 0.145 | 1.96 (1.38-2.79) | 0.00018 |
| rs12452392 | 17 | *(MYH2)* | Intergenic | G>A | 0.450 | 0.153 | 0.099 | 2.21 (1.47-3.32) | 0.00018 |
| rs2041127 | 17 | *(MYH3)* | Intergenic | G>A | 0.450 | 0.153 | 0.099 | 2.21 (1.47-3.32) | 0.00018 |
| rs7185024 | 16 | *(CDH11)* | Intergenic | A>C | 0.696 | 0.052 | 0.102 | 0.37 (0.21-0.65) | 0.00018 |
| rs995361 | 17 | *(MYH2)* | Intergenic | T>C | 0.581 | 0.208 | 0.142 | 1.99 (1.39-2.84) | 0.00018 |

Association analyses were adjusted by age, sex, and smoking status as covariates.

**P*-value of Hardy-Weinberg equilibrium (HWE).

Positive-specific IgE (D.p.) includes asthmatics showing the semi-quantitatively expressed concentration as class 3-6 (*n* = 173); negative as class 0 (*n* = 548).

Chr, chromosome; MAF, minor allele frequency; OR, odds ratio; CI, confidence interval.
