# Supplementary material for: A Genome-Wide Association Study of Total Serum and Mite-Specific IgEs in Asthma Patients
Source: PLoS One. 2013 Aug 13;8(8):e71958. doi: 10.1371/journal.pone.0071958 (PMC3742455; doi:10.1371/journal.pone.0071958)
Supplement: Table S4 — Top 100 SNPs associated with specific IgE for D.f. in the GWAS. (DOC) [file pone.0071958.s010.doc]

**Table S4.** Top 100 SNPs associated with specific IgE for D.f. in the GWAS

|  |  |  |  |  |  | MAF | |  |  |
| --- | --- | --- | --- | --- | --- | --- | --- | --- | --- |
|  |  |  |  |  |  | Specific IgE (D.f.) | |  |  |
| SNP ID | Chr | (Nearby) gene | Location | Variation | HWE* | Positive | Negative | OR (95% CI) | *P*-value |
| rs10142119 | 14 | *(LOC730217)* | Intergenic | G>A | 0.394 | 0.336 | 0.484 | 0.51 (0.39-0.66) | 1.86E-07 |
| rs1456988 | 14 | *(LOC730217)* | Intergenic | T>G | 0.475 | 0.580 | 0.450 | 1.86 (1.44-2.40) | 1.11E-06 |
| rs12590389 | 14 | *(LOC730217)* | Intergenic | T>C | 0.969 | 0.491 | 0.368 | 1.85 (1.43-2.40) | 2.47E-06 |
| rs6563898 | 16 | *CDH13* | Intron | G>A | 0.147 | 0.384 | 0.477 | 0.55 (0.42-0.72) | 8.19E-06 |
| rs132470 | 22 | *ARHGAP8* | Intron | T>C | 0.364 | 0.283 | 0.179 | 1.93 (1.44-2.61) | 1.13E-05 |
| rs4776381 | 15 | *(LBXCOR1)* | Intergenic | G>A | 0.140 | 0.168 | 0.081 | 2.26 (1.56-3.28) | 1.54E-05 |
| rs13274039 | 8 | *(LOC729478)* | Intergenic | G>A | 0.316 | 0.525 | 0.415 | 1.72 (1.34-2.21) | 1.56E-05 |
| rs2383513 | 1 | *(PTGS2)* | Intergenic | T>C | 0.744 | 0.550 | 0.442 | 1.75 (1.35-2.27) | 1.73E-05 |
| rs4761051 | 12 | *(TMEM132C)* | Intergenic | G>A | 0.756 | 0.475 | 0.378 | 1.73 (1.34-2.25) | 2.68E-05 |
| rs2273623 | 14 | *(PPM1A)* | Intergenic | A>G | 0.545 | 0.141 | 0.075 | 2.41 (1.59-3.65) | 3.16E-05 |
| rs12952334 | 17 | *MYH2* | Intron | G>A | 0.693 | 0.180 | 0.108 | 2.15 (1.49-3.09) | 3.29E-05 |
| rs10489397 | 1 | *(PTGS2)* | Intergenic | C>A | 0.885 | 0.541 | 0.434 | 1.72 (1.32-2.22) | 3.31E-05 |
| rs7434739 | 4 | *(PET112L)* | Intergenic | G>A | 0.705 | 0.252 | 0.348 | 0.56 (0.42-0.74) | 3.54E-05 |
| rs2852474 | 12 | *(KRT7)* | Intergenic | G>A | 0.861 | 0.093 | 0.190 | 0.46 (0.31-0.68) | 3.80E-05 |
| rs9810161 | 3 | *(LOC728135)* | Intergenic | G>A | 0.838 | 0.227 | 0.333 | 0.55 (0.41-0.74) | 3.94E-05 |
| rs1218762 | 16 | *OR2C1* | Coding | G>A | 0.422 | 0.123 | 0.185 | 0.48 (0.33-0.69) | 4.95E-05 |
| rs2305744 | 19 | *HPN* | Intron | A>G | 0.328 | 0.318 | 0.207 | 1.84 (1.37-2.47) | 4.97E-05 |
| rs7188903 | 16 | *(OR2C1)* | Intergenic | G>A | 0.562 | 0.123 | 0.186 | 0.48 (0.33-0.69) | 5.01E-05 |
| rs10926189 | 1 | *(GNG4)* | Intergenic | C>T | 0.234 | 0.216 | 0.137 | 2.00 (1.43-2.81) | 5.20E-05 |
| rs9310315 | 3 | *(LOC728135)* | Intergenic | T>C | 0.847 | 0.239 | 0.341 | 0.56 (0.42-0.75) | 5.26E-05 |
| rs12024772 | 1 | *HIVEP3* | Intron | G>A | 0.980 | 0.425 | 0.519 | 0.59 (0.46-0.77) | 5.73E-05 |
| rs6720449 | 2 | *(ARPC2)* | Intergenic | G>A | 0.821 | 0.218 | 0.304 | 0.55 (0.41-0.75) | 5.73E-05 |
| rs6052862 | 20 | *(RASSF2)* | Intergenic | T>G | 0.497 | 0.288 | 0.177 | 1.83 (1.36-2.46) | 5.91E-05 |
| rs2993120 | 1 | *HIVEP3* | Intron | A>C | 0.999 | 0.434 | 0.526 | 0.59 (0.46-0.77) | 6.00E-05 |
| rs2322732 | 11 | *(PRR10)* | Intergenic | G>A | 0.292 | 0.077 | 0.141 | 0.44 (0.29-0.67) | 6.07E-05 |
| rs2108606 | 17 | *MYH4* | Intron | T>C | 0.563 | 0.175 | 0.106 | 2.10 (1.46-3.02) | 6.18E-05 |
| rs941304 | 3 | *(CNTN3)* | Intergenic | A>G | 0.148 | 0.236 | 0.335 | 0.57 (0.43-0.76) | 6.24E-05 |
| rs7194029 | 16 | *CDH13* | Intron | C>T | 0.207 | 0.548 | 0.465 | 1.70 (1.30-2.21) | 6.25E-05 |
| rs2333166 | 4 | *(LOC391718)* | Intergenic | T>C | 0.719 | 0.061 | 0.128 | 0.41 (0.25-0.65) | 6.37E-05 |
| rs6496137 | 15 | *AKAP13* | Intron | A>C | 0.063 | 0.284 | 0.392 | 0.59 (0.45-0.77) | 6.39E-05 |
| rs4677485 | 3 | *(LOC728135)* | Intergenic | T>C | 0.645 | 0.218 | 0.321 | 0.56 (0.42-0.75) | 6.86E-05 |
| rs7196196 | 16 | *(OR2C1)* | Intergenic | A>C | 0.662 | 0.216 | 0.296 | 0.56 (0.41-0.75) | 6.92E-05 |
| rs11210537 | 1 | *HIVEP3* | Intron | A>G | 0.970 | 0.436 | 0.526 | 0.60 (0.46-0.77) | 6.99E-05 |
| rs763727 | 16 | *CDH13* | Intron | G>A | 0.423 | 0.439 | 0.520 | 1.68 (1.30-2.19) | 7.15E-05 |
| rs4140432 | 1 | *(PTGS2)* | Intergenic | T>G | 0.967 | 0.523 | 0.422 | 1.68 (1.29-2.17) | 7.28E-05 |
| rs4674267 | 2 | *(IL8RA)* | Intergenic | C>T | 0.615 | 0.234 | 0.314 | 0.56 (0.42-0.75) | 7.29E-05 |
| rs1035569 | 16 | *CDH13* | Intron | G>A | 0.206 | 0.545 | 0.464 | 1.69 (1.30-2.20) | 7.34E-05 |
| rs2286992 | 7 | *CPVL* | Intron | C>T | 0.394 | 0.268 | 0.376 | 0.58 (0.44-0.76) | 7.39E-05 |
| rs6571636 | 14 | *(C14orf147)* | Intergenic | G>A | 0.505 | 0.255 | 0.345 | 0.58 (0.43-0.76) | 8.05E-05 |
| rs1424169 | 16 | *CDH13* | Intron | G>A | 0.629 | 0.098 | 0.039 | 2.71 (1.65-4.47) | 8.42E-05 |
| rs10498606 | 14 | *(LOC283585)* | Intergenic | C>A | 0.504 | 0.543 | 0.414 | 1.64 (1.28-2.11) | 8.56E-05 |
| rs1930149 | 10 | *PCDH15* | Intron | A>G | 0.394 | 0.416 | 0.334 | 1.67 (1.29-2.17) | 8.75E-05 |
| rs1021891 | 17 | *(ACCN1)* | Intergenic | G>A | 0.011 | 0.282 | 0.191 | 1.86 (1.36-2.54) | 8.94E-05 |
| rs12670165 | 7 | *CPVL* | Intron | C>T | 0.426 | 0.268 | 0.375 | 0.58 (0.44-0.77) | 8.94E-05 |
| rs1566869 | 12 | *(ANKRD33)* | Intergenic | A>G | 0.615 | 0.409 | 0.308 | 1.68 (1.29-2.19) | 9.05E-05 |
| rs10261639 | 7 | *(EIF3IP1)* | Intergenic | T>G | 0.735 | 0.234 | 0.142 | 1.90 (1.38-2.63) | 9.26E-05 |
| rs1843834 | 2 | *(DOCK10)* | Intergenic | G>A | 0.478 | 0.266 | 0.181 | 1.81 (1.34-2.43) | 9.31E-05 |
| rs2820406 | 6 | *(ARID1B)* | Intergenic | G>A | 0.623 | 0.559 | 0.465 | 0.61 (0.47-0.78) | 9.53E-05 |
| rs17028407 | 4 | *(PET112L)* | Intergenic | C>A | 0.111 | 0.098 | 0.162 | 0.48 (0.32-0.71) | 9.59E-05 |
| rs2497708 | 10 | *ABLIM1* | Intron | C>A | 0.607 | 0.395 | 0.301 | 1.68 (1.29-2.18) | 0.00010 |
| rs8039009 | 15 | *LRRC28* | Intron | C>T | 0.861 | 0.127 | 0.070 | 2.30 (1.51-3.50) | 0.00010 |
| rs723919 | 16 | *CDH13* | Intron | G>A | 0.337 | 0.434 | 0.516 | 1.66 (1.28-2.16) | 0.00011 |
| rs7774434 | 6 | *(HLA-DQB1)* | Intergenic | T>C | 0.039 | 0.391 | 0.483 | 0.59 (0.45-0.78) | 0.00011 |
| rs6563897 | 16 | *CDH13* | Intron | G>A | 0.196 | 0.414 | 0.493 | 0.60 (0.46-0.78) | 0.00011 |
| rs628527 | 4 | *(LOC729265)* | Intergenic | A>G | 0.738 | 0.534 | 0.414 | 1.63 (1.27-2.09) | 0.00011 |
| rs338544 | 15 | *AKAP13* | Intron | T>C | 0.068 | 0.275 | 0.379 | 0.60 (0.46-0.78) | 0.00011 |

**Table S4.** Continued

| rs6717059 | 2 | *DOCK10* | Intron | T>C | 0.048 | 0.259 | 0.182 | 1.87 (1.36-2.58) | 0.00012 |
| --- | --- | --- | --- | --- | --- | --- | --- | --- | --- |
| rs12630469 | 3 | *(CNTN3)* | Intergenic | A>G | 0.642 | 0.280 | 0.392 | 0.59 (0.45-0.78) | 0.00012 |
| rs4590517 | 9 | *C9orf25* | Intron | C>T | 0.363 | 0.175 | 0.098 | 2.02 (1.41-2.89) | 0.00013 |
| rs3736388 | 3 | *(KALRN)* | Intergenic | T>C | 0.113 | 0.255 | 0.375 | 0.60 (0.46-0.78) | 0.00013 |
| rs40222 | 16 | *ZNF597* | Intron | T>C | 0.696 | 0.125 | 0.182 | 0.51 (0.36-0.73) | 0.00014 |
| rs1350279 | 14 | *(LOC730217)* | Intergenic | T>C | 0.160 | 0.325 | 0.420 | 0.61 (0.47-0.79) | 0.00014 |
| rs338540 | 15 | *AKAP13* | Intron | T>C | 0.077 | 0.277 | 0.379 | 0.60 (0.46-0.79) | 0.00015 |
| rs6713364 | 2 | *(LOC284998)* | Intergenic | T>C | 0.648 | 0.405 | 0.309 | 1.67 (1.28-2.17) | 0.00015 |
| rs10197382 | 2 | *(DOCK10)* | Intergenic | C>T | 0.137 | 0.314 | 0.229 | 1.76 (1.31-2.35) | 0.00015 |
| rs9604967 | 22 | *psiTPTE22* | Intron | C>T | 0.136 | 0.086 | 0.137 | 0.45 (0.29-0.70) | 0.00015 |
| rs9490054 | 6 | *(C6orf170)* | Intergenic | G>T | 0.782 | 0.166 | 0.106 | 2.02 (1.40-2.91) | 0.00015 |
| rs10410631 | 19 | *ZNF550* | Coding | C>T | 0.898 | 0.373 | 0.464 | 0.61 (0.48-0.79) | 0.00015 |
| rs4812609 | 20 | *PTPRT* | Intron | A>G | 0.680 | 0.559 | 0.462 | 1.61 (1.25-2.07) | 0.00016 |
| rs2156208 | 18 | *(LOC390861)* | Intergenic | T>C | 0.942 | 0.102 | 0.051 | 2.51 (1.56-4.04) | 0.00016 |
| rs446924 | 1 | *ABCB10* | Intron | C>T | 0.028 | 0.064 | 0.124 | 0.44 (0.28-0.70) | 0.00016 |
| rs2489382 | 10 | *C10orf141* | Intron | G>A | 0.855 | 0.423 | 0.524 | 0.62 (0.48-0.80) | 0.00016 |
| rs13099862 | 3 | *(CNTN3)* | Intergenic | T>C | 0.273 | 0.220 | 0.311 | 0.59 (0.44-0.78) | 0.00017 |
| rs9828201 | 3 | *(LOC728135)* | Intergenic | T>C | 0.898 | 0.305 | 0.429 | 0.61 (0.46-0.79) | 0.00017 |
| rs2244811 | 17 | *(NXN)* | Intergenic | A>G | 0.224 | 0.141 | 0.080 | 2.22 (1.46-3.37) | 0.00017 |
| rs12458013 | 18 | *(SMAD2)* | Intergenic | A>G | 0.907 | 0.100 | 0.173 | 0.49 (0.33-0.72) | 0.00017 |
| rs8035496 | 15 | *AKAP13* | Intron | T>C | 0.070 | 0.280 | 0.381 | 0.61 (0.47-0.79) | 0.00018 |
| rs11209099 | 1 | *(SERBP1)* | Intergenic | G>A | 0.007 | 0.270 | 0.354 | 0.61 (0.47-0.79) | 0.00018 |
| rs2428684 | 17 | *ACSF2* | Intron | C>A | 0.660 | 0.368 | 0.268 | 1.67 (1.28-2.19) | 0.00018 |
| rs6585041 | 10 | *(ADRA2A)* | Intergenic | T>C | 0.810 | 0.386 | 0.478 | 0.62 (0.48-0.80) | 0.00018 |
| rs40633 | 16 | *(ZNF434)* | Intergenic | A>G | 0.997 | 0.118 | 0.174 | 0.50 (0.34-0.73) | 0.00018 |
| rs12046472 | 1 | *(PLD5)* | Intergenic | G>A | 0.266 | 0.130 | 0.061 | 2.25 (1.47-3.44) | 0.00018 |
| rs11203582 | 8 | *SGCZ* | Intron | G>A | 0.657 | 0.293 | 0.384 | 0.60 (0.46-0.79) | 0.00018 |
| rs17609347 | 16 | *(LOC645478)* | Intergenic | C>A | 0.669 | 0.126 | 0.068 | 2.26 (1.48-3.44) | 0.00018 |
| rs1925227 | 6 | *GRIK2* | Intron | C>T | 0.269 | 0.105 | 0.182 | 0.51 (0.35-0.74) | 0.00018 |
| rs6790277 | 3 | *(CNTN3)* | Intergenic | G>T | 0.256 | 0.220 | 0.310 | 0.59 (0.44-0.78) | 0.00019 |
| rs956706 | 1 | *SMYD3* | Intron | C>T | 0.597 | 0.281 | 0.192 | 1.73 (1.30-2.32) | 0.00019 |
| rs4420916 | 3 | *(CNTN3)* | Intergenic | G>A | 0.497 | 0.284 | 0.393 | 0.60 (0.46-0.79) | 0.00019 |
| rs6795835 | 3 | *(LOC728135)* | Intergenic | C>T | 0.497 | 0.284 | 0.393 | 0.60 (0.46-0.79) | 0.00019 |
| rs7629942 | 3 | *(CNTN3)* | Intergenic | G>A | 0.497 | 0.284 | 0.393 | 0.60 (0.46-0.79) | 0.00019 |
| rs12469757 | 2 | *(DOCK10)* | Intergenic | G>A | 0.125 | 0.298 | 0.220 | 1.76 (1.30-2.37) | 0.00019 |
| rs987651 | 5 | *STK32A* | Intron | C>T | 0.302 | 0.552 | 0.464 | 1.62 (1.25-2.10) | 0.00019 |
| rs17065935 | 8 | *CSMD1* | Intron | A>G | 0.073 | 0.136 | 0.228 | 0.54 (0.39-0.76) | 0.00019 |
| rs7144501 | 14 | *(LOC730217)* | Intergenic | G>A | 0.838 | 0.239 | 0.328 | 0.59 (0.44-0.78) | 0.00020 |
| rs10014970 | 4 | *(ANTXR2)* | Intergenic | G>A | 0.426 | 0.339 | 0.248 | 1.70 (1.28-2.25) | 0.00020 |
| rs7905868 | 10 | *(FAM107B)* | Intergenic | T>C | 0.260 | 0.323 | 0.233 | 1.68 (1.28-2.21) | 0.00021 |
| rs7793513 | 7 | *(RAPGEF5)* | Intergenic | T>C | 0.888 | 0.534 | 0.433 | 1.60 (1.25-2.06) | 0.00021 |
| rs10204762 | 2 | *(LOC284998)* | Intergenic | G>A | 0.544 | 0.220 | 0.148 | 1.85 (1.34-2.56) | 0.00021 |
| rs2383477 | 1 | *C1orf27* | Intron | C>T | 0.128 | 0.032 | 0.088 | 0.34 (0.18-0.64) | 0.00021 |
| rs1565073 | 2 | *DOCK10* | Intron | G>T | 0.037 | 0.284 | 0.206 | 1.79 (1.31-2.44) | 0.00022 |

Association analyses were adjusted by age, sex, and smoking status as covariates.

**P*-value of Hardy-Weinberg equilibrium (HWE).

Positive-specific IgE (D.f.) includes asthmatics showing the semi-quantitatively expressed concentration as class 3-6 (*n* = 220); negative as class 0 (*n* = 473).

Chr, chromosome; MAF, minor allele frequency; OR, odds ratio; CI, confidence interval.
