# Supplementary material for: A Genome-Wide Association Study of Total Serum and Mite-Specific IgEs in Asthma Patients
Source: PLoS One. 2013 Aug 13;8(8):e71958. doi: 10.1371/journal.pone.0071958 (PMC3742455; doi:10.1371/journal.pone.0071958)
Supplement: Table S5 — Pathway analysis for significantly associated SNPs ( P <0.001), only in the gene regions from the results of GWAS, on total IgE in asthmatics. (DOC) [file pone.0071958.s011.doc]

**Table S5.** Pathway analysis for significantly associated SNPs (*P* < 0.001), only in the gene regions from the results of GWAS, on total IgE in asthmatics

| Pathway Name | Impact Factor | #Genes in Pathway | #Input Genes in Pathway | #Pathway Genes on Chip | Corrected gamma *P*-value |
| --- | --- | --- | --- | --- | --- |
| Phosphatidylinositol signaling system | 44.198 | 76 | 2 | 76 | 2.89E-18 |
| Adherens junction | 33.006 | 78 | 2 | 75 | 1.57E-13 |
| Focal adhesion | 8.87 | 203 | 5 | 199 | 0.001 |
| Glioma | 8.406 | 65 | 3 | 65 | 0.002 |
| MAPK signaling pathway | 7.516 | 272 | 1 | 266 | 0.005 |
| Type II diabetes mellitus | 7.436 | 45 | 1 | 43 | 0.005 |
| Insulin signaling pathway | 6.6 | 138 | 1 | 137 | 0.01 |
| Melanoma | 6.37 | 71 | 1 | 71 | 0.01 |
| Long-term depression | 6.066 | 75 | 2 | 73 | 0.02 |
| Long-term potentiation | 5.919 | 73 | 2 | 72 | 0.02 |
| Natural killer cell mediated cytotoxicity | 5.853 | 135 | 2 | 130 | 0.02 |
| Regulation of actin cytoskeleton | 5.839 | 217 | 3 | 207 | 0.02 |
| Calcium signaling pathway | 5.48 | 182 | 3 | 176 | 0.03 |
| Prostate cancer | 5.353 | 90 | 1 | 89 | 0.03 |
| ErbB signaling pathway | 4.825 | 87 | 2 | 86 | 0.05 |
| Jak-STAT signaling pathway | 4.587 | 155 | 1 | 153 | 0.06 |
| Maturity onset diabetes of the young | 4.448 | 24 | 1 | 23 | 0.06 |
| Cardiac muscle contraction | 4.332 | 87 | 2 | 73 | 0.07 |
| Axon guidance | 4.209 | 129 | 1 | 128 | 0.08 |
| GnRH signaling pathway | 3.751 | 103 | 2 | 100 | 0.11 |
| ECM-receptor interaction | 3.197 | 84 | 1 | 82 | 0.17 |
| Vibrio cholerae infection | 3.058 | 62 | 1 | 59 | 0.19 |
| Type I diabetes mellitus | 2.863 | 44 | 1 | 40 | 0.22 |
| Pathways in cancer | 2.543 | 330 | 2 | 328 | 0.28 |
| Olfactory transduction | 2.505 | 382 | 1 | 374 | 0.29 |
| Neuroactive ligand-receptor interaction | 2.415 | 256 | 2 | 250 | 0.31 |
| Chronic myeloid leukemia | 2.319 | 75 | 1 | 74 | 0.33 |
| Colorectal cancer | 2.305 | 84 | 1 | 83 | 0.33 |
| Cytokine-cytokine receptor interaction | 2.258 | 263 | 2 | 259 | 0.34 |
| Small cell lung cancer | 2.198 | 86 | 1 | 85 | 0.36 |
| TGF-beta signaling pathway | 2.198 | 87 | 1 | 85 | 0.36 |
| Melanogenesis | 2.053 | 102 | 1 | 102 | 0.39 |
| Leukocyte transendothelial migration | 1.987 | 119 | 1 | 115 | 0.41 |
| Cell adhesion molecules (CAMs) | 1.898 | 134 | 1 | 129 | 0.43 |
| Wnt signaling pathway | 1.756 | 152 | 1 | 150 | 0.48 |
| Alzheimer''s disease | 1.719 | 178 | 1 | 159 | 0.49 |

Associated pathway with total IgE in asthmatics is estimated using Pathway Express (<http://vortex.cs.wayne.edu/projects.htm>).
