# Supplementary material for: A Genome-Wide Association Study of Total Serum and Mite-Specific IgEs in Asthma Patients
Source: PLoS One. 2013 Aug 13;8(8):e71958. doi: 10.1371/journal.pone.0071958 (PMC3742455; doi:10.1371/journal.pone.0071958)
Supplement: Table S6 — Statistical power for the analysis in each subgroup of study subjects. (DOC) [file pone.0071958.s012.doc]

**Table S6.** Statistical power for the analysis in each subgroup of study subjects

| Analysis group | Total IgE | Specific IgE (D.p.) | Specific IgE (D.f.) |
| --- | --- | --- | --- |
| Sample size | n = 877 | Positive (n = 173) vs. | Positive (n = 220) vs. |
|  |  | Negative (n = 548) | Negative (n = 473) |
| Statistical power | 44.5% | 92.0% | 62.0% |

Statistical power of linear regression analysis for total IgE is obtained from two-tailed *t*-test.

Statistical power of case-control analysis for specific IgE is obtained using the CaTS power calculator (Skol et al., 2006).
