# Supplementary material for: A Genome-Wide Association Study of Total Serum and Mite-Specific IgEs in Asthma Patients
Source: PLoS One. 2013 Aug 13;8(8):e71958. doi: 10.1371/journal.pone.0071958 (PMC3742455; doi:10.1371/journal.pone.0071958)
Supplement: Table S7 — Genetic associations from this GWAS compared to the previously identified SNPs from other GWASs. (DOC) [file pone.0071958.s013.doc]

**Table S7.** Genetic associations from this GWAS compared to the previously identified SNPs from other GWASs

|  |  |  | This study | | Previous GWASs |
| --- | --- | --- | --- | --- | --- |
| Gene | SNP ID | Chr | MAF | *P*-value | *P*-value |
| *FCER1A* | rs2511211 | 1 | 0.000** | N/A | 9.28×10^-8^ [ref. 5] |
| *FCER1A* | rs10489854 | 1 | 0.460 | 0.51 | 0.029 [ref. 5] |
| *FCER1A* | rs2494262 | 1 | - | N/A | 1.67×10^-4^ [ref. 5] |
| *FCER1A* | rs2427837* | 1 | 0.035 | 0.62 | 3.23×10^-9^ [ref. 5] |
| *FCER1A* | rs12565775* | 1 | 0.425 | 0.36 | 0.025 [ref. 5] |
| *FCER1A* | rs2427824 | 1 | 0.160 | 0.46 | 0.025 [ref. 5] |
| *FCER1A* | rs2427827* | 1 | 0.190 | 0.29 | 2.45×10^-4^ [ref. 5] |
| *FCER1A* | rs2251746 | 1 | 0.035 | 0.62 | 6.07×10^-10^ [ref. 5]; 2.11×10^-12^ [ref. 7]; 0.0072 [ref. 8] |
| *FCER1A* | rs2252226 | 1 | 0.190 | 0.29 | 6.60×10^-5^ [ref. 6] |
| *FCER1A* | rs2494264 | 1 | - | N/A | 1.37×10^-9^ [ref. 7]; 0.235 [ref. 8] |
| *FCER1A* | rs2252226 | 1 | 0.190 | 0.29 | 0.0060 [ref. 8] |
| *RAD50* | rs2706347* | 5 | 0.204 | 0.16 | 2.26×10^-4^ [ref. 5]; 0.0064 [ref. 8] |
| *RAD50* | rs3798135* | 5 | 0.205 | 0.17 | 2.32×10^-4^ [ref. 5]; 8.38×10^-5^ [ref. 8] |
| *RAD50* | rs2040704* | 5 | 0.205 | 0.17 | 2.47×10^-4^ [ref. 5]; 0.0007 [ref. 8] |
| *RAD50* | rs7737470* | 5 | 0.205 | 0.17 | 2.27×10^-4^ [ref. 5]; 0.021[ref. 8] |
| *RAD50* | rs2240032 | 5 | 0.205 | 0.17 | 4.01×10^-4^ [ref. 5] |
| *IL13* | rs20541 | 5 | 0.312 | 0.34 | 1.00×10^-6^ [ref. 6]; 3.55×10^-8^ [ref. 7] ; 0.025 [ref. 8] |
| *HLA-DRB1* | rs9271300 | 6 | - | N/A | 8.30×10^-15^ [ref. 6] |
| *STAT6* | rs12368672 | 12 | - | N/A | 2.03×10^-6^ [ref. 5]; 0.0022[ref. 8] |
| *STAT6* | rs167769 | 12 | 0.259 | 0.82 | 8.50×10^-7^ [ref. 6]; 5.18×10^-6^ [ref. 7]; 2.48×10^-5^ [ref. 8] |
| *STAT6* | rs1059513 | 12 | 0.067 | 0.077 | 2.87×10^-8^ [ref. 7] |
| *IL4R/IL21R* | rs1859308 | 16 | 0.288 | 0.59 | 8.20×10^-6^ [ref. 6]; 0.22[ref. 8] |

*Imputed SNP based on nearby SNPs with absolute LD (|*D'*| = 1 and *r^2^* = 1).

**MAF of Asians from dbSNP of the National Center for Biotechnology Information (http://www.ncbi.nlm.nih.gov/snp).

N/A, not available due to no nearby SNPs with tight LD (*r^2^* > 0.8) or monomorphic allele.

Chr, chromosome; MAF, minor allele frequency; LD, linkage disequilibrium.
