# Supplementary material for: A Genome-Wide Association Study of Total Serum and Mite-Specific IgEs in Asthma Patients
Source: PLoS One. 2013 Aug 13;8(8):e71958. doi: 10.1371/journal.pone.0071958 (PMC3742455; doi:10.1371/journal.pone.0071958)
Supplement: Table S8 — SNPs of other potential genes (but not well-known IgE-influencing genes) associated with total IgE in asthmatics ( P <0.01). (DOC) [file pone.0071958.s014.doc]

**Table S8.** SNPs of other potential genes (but not well-known IgE-influencing genes) associated with total IgE in asthmatics (*P* < 0.01)

|  |  |  |  |  |  |  |  | Genotype** | | |  |  |
| --- | --- | --- | --- | --- | --- | --- | --- | --- | --- | --- | --- | --- |
| SNP ID | Chr | Gene | Location | AA change | Variation | MAF | HWE* | M/M | M/m | m/m | *P*-value† | *P*-value†† |
| rs1006393 | 7 | *NPSR1* | Flanking_5'UTR |  | T>C | 0.298 | 0.052 | 444 (2.21) | 343 (2.13) | 90 (2.21) | 0.37 | 0.59 |
| rs376825 | 7 | *NPSR1* | Flanking_5'UTR |  | A>C | 0.297 | 0.043 | 445 (2.21) | 341 (2.13) | 90 (2.21) | 0.40 | 0.64 |
| rs1458550 | 7 | *NPSR1* | Flanking_5'UTR |  | G>A | 0.152 | 0.336 | 634 (2.17) | 219 (2.20) | 24 (2.17) | 0.98 | 0.96 |
| rs1023556 | 7 | *NPSR1* | Flanking_5'UTR |  | C>T | 0.454 | 0.579 | 257 (2.19) | 443 (2.17) | 177 (2.18) | 0.71 | 0.69 |
| rs2530545 | 7 | *NPSR1* | Flanking_5'UTR |  | C>T | 0.177 | 0.086 | 601 (2.21) | 241 (2.08) | 35 (2.40) | 0.54 | 0.91 |
| rs887020 | 7 | *NPSR1* | 5'UTR |  | G>A | 0.225 | 0.386 | 522 (2.19) | 315 (2.19) | 40 (1.99) | 0.40 | 0.51 |
| rs2530548 | 7 | *NPSR1* | Intron |  | A>G | 0.136 | 0.415 | 657 (2.21) | 200 (2.08) | 19 (2.11) | **0.03** | **0.008** |
| rs2530552 | 7 | *NPSR1* | Intron |  | A>G | 0.136 | 0.436 | 657 (2.21) | 201 (2.08) | 19 (2.11) | **0.02** | **0.007** |
| rs11761197 | 7 | *NPSR1* | Intron |  | A>G | 0.117 | 0.995 | 684 (2.16) | 181 (2.25) | 12 (2.09) | 0.11 | 0.05 |
| rs1379928 | 7 | *NPSR1* | Intron |  | T>C | 0.205 | 0.326 | 550 (2.19) | 295 (2.15) | 32 (2.26) | 0.85 | 1.00 |
| rs411323 | 7 | *NPSR1* | Intron |  | C>T | 0.198 | 0.358 | 560 (2.16) | 287 (2.22) | 30 (2.19) | 0.33 | 0.25 |
| rs425990 | 7 | *NPSR1* | Intron |  | T>C | 0.251 | 0.991 | 487 (2.19) | 327 (2.17) | 55 (2.11) | 0.64 | 0.54 |
| rs2530561 | 7 | *NPSR1* | Intron |  | C>T | 0.088 | 0.456 | 727 (2.20) | 144 (2.07) | 5 (2.45) | 0.12 | **0.02** |
| rs2609224 | 7 | *NPSR1* | Intron |  | T>G | 0.202 | 0.749 | 554 (2.18) | 284 (2.17) | 34 (2.24) | 0.66 | 0.77 |
| rs2609220 | 7 | *NPSR1* | Intron |  | G>A | 0.196 | 0.710 | 565 (2.18) | 280 (2.17) | 32 (2.26) | 0.60 | 0.68 |
| rs2530571 | 7 | *NPSR1* | Intron |  | G>A | 0.237 | 0.264 | 515 (2.19) | 304 (2.19) | 55 (2.11) | 0.84 | 0.73 |
| rs10486653 | 7 | *NPSR1* | Intron |  | C>T | 0.049 | 0.937 | 793 (2.19) | 82 (2.11) | 2 (1.62) | 0.17 | 0.19 |
| rs1458536 | 7 | *NPSR1* | Intron |  | G>A | 0.080 | 0.787 | 742 (2.19) | 130 (2.09) | 5 (2.45) | 0.27 | 0.09 |
| rs2609215 | 7 | *NPSR1* | Intron |  | A>G | 0.080 | 0.787 | 742 (2.19) | 130 (2.09) | 5 (2.45) | 0.27 | 0.09 |
| rs10255979 | 7 | *NPSR1* | Intron |  | C>T | 0.450 | 0.315 | 270 (2.13) | 415 (2.22) | 183 (2.15) | 0.48 | 0.45 |
| rs2531840 | 7 | *NPSR1* | Intron |  | G>A | 0.129 | 0.182 | 670 (2.20) | 188 (2.10) | 19 (2.11) | 0.09 | **0.03** |
| rs2531841 | 7 | *NPSR1* | Intron |  | T>C | 0.245 | 0.532 | 502 (2.19) | 317 (2.18) | 56 (2.10) | 0.78 | 0.73 |
| rs10274146 | 7 | *NPSR1* | Intron |  | A>G | 0.049 | 0.937 | 793 (2.19) | 82 (2.11) | 2 (1.62) | 0.17 | 0.19 |
| rs1419837 | 7 | *NPSR1* | Flanking_3'UTR |  | C>T | 0.116 | 0.964 | 685 (2.16) | 180 (2.25) | 12 (2.09) | 0.12 | 0.05 |
| rs1436849 | 12 | *IRAK3* | Flanking_5'UTR |  | T>C | 0.226 | 0.710 | 523 (2.23) | 311 (2.14) | 43 (1.93) | **0.004** | **0.01** |
| rs2701653 | 12 | *IRAK3* | Flanking_5'UTR |  | C>T | 0.420 | 0.763 | 293 (2.09) | 431 (2.22) | 152 (2.24) | 0.02 | **0.02** |
| rs1732877 | 12 | *IRAK3* | Intron |  | C>T | 0.452 | 0.434 | 269 (2.19) | 423 (2.22) | 185 (2.08) | 0.13 | 0.27 |
| rs1152888 | 12 | *IRAK3* | Coding | I147V | G>A | 0.404 | 0.644 | 308 (2.10) | 429 (2.20) | 140 (2.28) | **0.003** | **0.007** |
| rs1821777 | 12 | *IRAK3* | Intron |  | C>T | 0.142 | 0.643 | 644 (2.20) | 217 (2.14) | 16 (1.94) | 0.05 | **0.03** |
| rs1623665 | 12 | *IRAK3* | Intron |  | T>G | 0.450 | 0.303 | 273 (2.18) | 419 (2.23) | 185 (2.08) | 0.26 | 0.44 |
| rs17826057 | 12 | *IRAK3* | Intron |  | G>A | 0.052 | 0.804 | 788 (2.18) | 87 (2.17) | 2 (1.98) | 0.77 | 0.67 |
| rs1152912 | 12 | *IRAK3* | Intron |  | G>A | 0.355 | 0.958 | 365 (2.19) | 401 (2.16) | 111 (2.20) | 0.65 | 0.64 |
| rs1152918 | 12 | *IRAK3* | Intron |  | C>T | 0.405 | 0.604 | 307 (2.14) | 430 (2.22) | 140 (2.13) | 0.83 | 0.64 |
| rs3782348 | 12 | *IRAK3* | Intron |  | C>T | 0.405 | 0.664 | 307 (2.14) | 429 (2.22) | 141 (2.13) | 0.88 | 0.69 |
| rs12307857 | 12 | *IRAK3* | Flanking_3'UTR |  | T>C | 0.270 | 0.613 | 470 (2.18) | 340 (2.18) | 67 (2.18) | 0.47 | 0.40 |

**P*-value of Hardy-Weinberg equilibrium (HWE).

**Genotype represents number of subjects (mean of Log[Total IgE (IU/ml)]).

M/M, M/m, and m/m indicate homozygote for common allele, heterozygote, and homozygote for rare allele, respectively.

†Association analyses were adjusted by age, sex, and smoking status as covariates.

††Association analyses were adjusted by age, sex, smoking status, and atopy as covariates

Chr, chromosome; AA, amino acid; MAF, minor allele frequency.
