# Supplementary material for: A Genome-Wide Association Study of Total Serum and Mite-Specific IgEs in Asthma Patients
Source: PLoS One. 2013 Aug 13;8(8):e71958. doi: 10.1371/journal.pone.0071958 (PMC3742455; doi:10.1371/journal.pone.0071958)
Supplement: Table S9 — Genes based clustering from GRAIL. (DOC) [file pone.0071958.s015.doc]

**Table S9.** Genes based clustering from GRAIL

| **Gene** | **GRAIL *P*-value** | **Selected similar genes (Rank in parantheses)** | | |  |  |  |  |  |  |  |  |  |  |
| --- | --- | --- | --- | --- | --- | --- | --- | --- | --- | --- | --- | --- | --- | --- |
| KALRN | **0.011** | SIPA1L1(14) | VAV2(150) | ARHGDIB(163) | RIN3(455) | PLXDC2(610) | CRIM1(806) | LSAMP(879) | A2BP1(1178) | BRD3(1210) | PIP3-E(1498) | IGF1R(1714) | UNC13C(1729) | NOL10(1816) |
| CNTN5 | **0.011** | PLXDC2(82) | LSAMP(99) | CRIM1(270) | KALRN(323) | A2BP1(443) | UNC13C(580) | CACNA1C(862) | CSMD1(1037) | SVOP(1545) | BRD3(1783) | NOL10(1923) |  |  |
| INPP4B | **0.015** | PIB5PA(15) | IHPK2(71) | IGF1R(332) | PTPRN2(356) | CSMD1(381) | PIP3-E(496) | PLXDC2(753) | SYNPO2(791) | CCDC85A(1572) | BRD3(1936) |  |  |  |
| SLC6A2 | **0.016** | ADRA1A(76) | SLC45A2(248) | CACNA1C(355) | PLXDC2(423) | A2BP1(450) | SEC24D(682) | OPRM1(966) | LSAMP(999) | KALRN(1031) | BRD3(1099) | CSMD1(1141) | CRIM1(1343) | IGF1R(1373) |
|  |  | SVOP(1542) | IHPK2(1770) | DGKH(1845) |  |  |  |  |  |  |  |  |  |  |
| UNC13C | **0.018** | KALRN(109) | SVOP(142) | SIPA1L1(163) | PLXDC2(504) | CRIM1(574) | LSAMP(829) | CNTN5(894) | CACNA1C(1112) | A2BP1(1119) | FAM62B(1211) | PTPRN2(1533) |  |  |
| LSAMP | **0.021** | PLXDC2(28) | CSMD1(89) | KALRN(166) | SVOP(492) | BRD3(717) | CRIM1(825) | NOL10(1091) | A2BP1(1266) | CNTN5(1730) |  |  |  |  |
| HPSE2 | **0.025** | CSMD1(14) | PLXDC2(206) | C1orf21(260) | ZEB2(304) | SYNPO2(401) | LSAMP(1192) | VPS13B(1194) | BRD3(1310) | SLC45A2(1519) | IGF1R(1822) | NOL10(1847) |  |  |
| ADRA1A | **0.027** | CACNA1C(22) | SLC6A2(98) | IGF1R(305) | LAMA1(459) | OPRM1(554) | PLXDC2(1223) | KALRN(1395) | IHPK2(1669) | LSAMP(1831) | SYNPO2(1945) |  |  |  |
| OPRM1 | **0.030** | ADRA1A(119) | SLC6A2(222) | IGF1R(295) | CACNA1C(447) | PLXDC2(643) | LSAMP(664) | KALRN(773) | A2BP1(1210) | BRD3(1233) | CRIM1(1690) | SLC45A2(1876) | CSMD1(1956) |  |
| VAV2 | **0.037** | KALRN(19) | ARHGDIB(56) | IGF1R(508) | PIP3-E(754) | IQGAP1(832) | RIN3(1169) | CRIM1(1344) | BRD3(1353) | LAMA1(1652) |  |  |  |  |
| ARHGDIB | 0.058 | KALRN(44) | VAV2(75) | SYNPO2(261) | CSMD1(658) | IGF1R(785) | IQGAP1(1053) | PIP3-E(1090) | PLXDC2(1508) | BRD3(1635) | CRIM1(1780) |  |  |  |
| VPS13B | 0.059 | ZEB2(18) | SLC45A2(181) | CSMD1(381) | NOL10(410) | PLXDC2(742) | HPSE2(1315) | TMEM180(1692) | C1orf21(1776) | A2BP1(1841) | SVOP(1878) |  |  |  |
| IHPK2 | 0.084 | PIB5PA(16) | INPP4B(47) | PLXDC2(818) | NOL10(887) | BRD3(1022) | SVOP(1212) | SHISA5(1273) | NUDCD3(1580) | IGF1R(1816) | GTDC1(1969) |  |  |  |
| IQGAP1 | 0.088 | KALRN(138) | ARHGDIB(195) | VAV2(290) | SYNPO2(349) | TLN1(817) | SIPA1L1(1099) | CACNA1C(1297) | IGF1R(1466) | LRRC16A(1472) | BRD3(1524) | PIP3-E(1591) |  |  |
| COL7A1 | 0.098 | LAMA1(103) | SLC45A2(115) | BRD3(551) | CSMD1(607) | NOL10(672) | ZEB2(741) | VPS13B(1094) | PLXDC2(1168) | CACNA1C(1833) |  |  |  |  |
| GALNT14 | 0.120 | IGF1R(37) | CSMD1(340) | SHISA5(544) | SYNPO2(563) | PLXDC2(877) | C1orf21(1572) | CRIM1(1678) | IHPK2(1720) | TMEM180(1989) |  |  |  |  |
| ADAM12 | 0.123 | IGF1R(108) | PLXDC2(351) | LAMA1(498) | CRIM1(634) | BRD3(771) | CSMD1(820) | KALRN(1398) | SYNPO2(1473) | CACNA1C(1521) | NOL10(1684) |  |  |  |
| ZEB2 | 0.123 | VPS13B(125) | CSMD1(178) | CRIM1(242) | PLXDC2(655) | BRD3(744) | NOL10(1481) | SLC45A2(1537) |  |  |  |  |  |  |
| PIB5PA | 0.142 | INPP4B(9) | IHPK2(512) | NOL10(705) | PLXDC2(907) | GTDC1(956) | SVOP(1069) | BRD3(1143) | NUDCD3(1343) | FAM62B(1887) |  |  |  |  |
| RIN3 | 0.191 | KALRN(27) | VAV2(425) | PIP3-E(735) | NOL10(762) | PLXDC2(821) | ARHGDIB(1057) | SIPA1L1(1229) | PIB5PA(1662) |  |  |  |  |  |
| NPC1L1 | 0.198 | OSBPL1A(162) | SLC6A2(488) | IGF1R(505) | IHPK2(724) | PLXDC2(1286) | CACNA1C(1421) | ADRA1A(1491) | SLC45A2(1566) | KALRN(1882) | FAM62B(1955) |  |  |  |
| ATP6V1C2 | 0.200 | SYT17(90) | CACNA1C(102) | BRD3(495) | FAM62B(1116) | LAMA1(1164) | PLXDC2(1458) | NOL10(1802) | CRIM1(1958) |  |  |  |  |  |
| SIPA1L1 | 0.226 | KALRN(14) | NOL10(576) | PLXDC2(804) | BRD3(873) | SVOP(1300) | UNC13C(1497) |  |  |  |  |  |  |  |
| CSMD1 | 0.233 | PLXDC2(112) | SYNPO2(223) | NOL10(287) | A2BP1(665) | LSAMP(1266) | BRD3(1284) | TMEM180(1948) |  |  |  |  |  |  |
| PTPRN2 | 0.238 | IGF1R(126) | PLXDC2(340) | KALRN(894) | PIB5PA(913) | INPP4B(936) | SVOP(1242) | CACNA1C(1543) | SYT17(1615) | BRD3(1845) |  |  |  |  |
| SLC45A2 | 0.260 | PLXDC2(294) | CSMD1(356) | NOL10(385) | BRD3(471) | SLC6A2(591) | A2BP1(1536) | VPS13B(1908) |  |  |  |  |  |  |
| CRIM1 | 0.305 | PLXDC2(43) | IGF1R(421) | BRD3(951) | TMEM180(1256) | KALRN(1262) | NOL10(1401) | SHISA5(1977) |  |  |  |  |  |  |
| PLA2G3 | 0.313 | CACNA1C(409) | PLXDC2(518) | CRIM1(595) | KALRN(818) | SHISA5(1051) | IGF1R(1224) | BRD3(1486) | PIB5PA(1716) | IHPK2(1757) | PLSCR1(1981) |  |  |  |
| A2BP1 | 0.329 | PLXDC2(141) | CSMD1(355) | NOL10(632) | CACNA1C(1234) | DGKH(1285) | BRD3(1373) | KALRN(1543) | TMEM180(1789) |  |  |  |  |  |
| TLN1 | 0.340 | LAMA1(199) | SYNPO2(384) | KALRN(481) | LRRC16A(810) | BRD3(1090) | PIB5PA(1301) | SIPA1L1(1365) | NOL10(1773) | IQGAP1(1922) | VAV2(1936) | CACNA1C(1950) |  |  |
| SYT17 | 0.355 | ATP6V1C2(104) | FAM62B(220) | KALRN(722) | CRIM1(1051) | CACNA1C(1100) | BRD3(1124) | PLXDC2(1534) | PTPRN2(1584) | PIB5PA(1713) |  |  |  |  |
| OSMR | 0.361 | IGF1R(160) | NLRP2(767) | PLXDC2(835) | CRIM1(1177) | LAMA1(1448) | BRD3(1518) | CPM(1599) | CACNA1C(1601) | CSMD1(1676) | ADRA1A(1996) |  |  |  |
| IGF1R | 0.368 | GALNT14(212) | CRIM1(253) | PTPRN2(982) | BRD3(1205) | PLXDC2(1236) | ADRA1A(1357) | KALRN(1420) | IHPK2(1697) | CACNA1C(1817) |  |  |  |  |
| PI16 | 0.445 | NOL10(239) | TMEM180(279) | PLXDC2(860) | BRD3(1057) | SVOP(1134) | IGF1R(1624) | CRIM1(1835) |  |  |  |  |  |  |
| LAMA1 | 0.452 | PLXDC2(241) | CRIM1(448) | BRD3(887) | ADRA1A(1046) | KALRN(1414) | CACNA1C(1608) | TLN1(1842) |  |  |  |  |  |  |
| PIP3-E | 0.457 | CCDC85A(192) | KALRN(650) | PLXDC2(781) | C1orf21(1113) | NOL10(1271) | BRD3(1871) | SYNPO2(1922) | CSMD1(1955) |  |  |  |  |  |
| SYNPO2 | 0.463 | CSMD1(93) | C1orf21(509) | BRD3(1175) | NOL10(1201) | PLXDC2(1204) | SHISA5(1944) |  |  |  |  |  |  |  |
| SBNO2 | 0.490 | PLXDC2(239) | BRD3(524) | CCDC85A(648) | CRIM1(1208) | NOL10(1345) | TMEM180(1425) |  |  |  |  |  |  |  |
| FAM62B | 0.519 | NOL10(479) | SVOP(585) | PLXDC2(706) | TMEM180(923) | NUDCD3(1081) | GTDC1(1085) | CCDC85A(1251) | C1orf21(1550) | BRD3(1820) |  |  |  |  |
| CACNA1C | 0.542 | ADRA1A(238) | A2BP1(708) | ATP6V1C2(1045) | PLXDC2(1362) | IGF1R(1410) | KALRN(1524) | SLC6A2(1691) | BRD3(1825) |  |  |  |  |  |
| PLSCR1 | 0.546 | CACNA1C(618) | PIB5PA(905) | BRD3(1022) | IHPK2(1034) | IGF1R(1052) | CRIM1(1138) | SHISA5(1147) | PLXDC2(1159) | LAMA1(1947) | SYNPO2(1967) |  |  |  |
| SEC24D | 0.557 | SLC6A2(335) | NOL10(742) | CCDC85A(966) | SVOP(1241) | PLXDC2(1460) | OSBPL1A(1480) | SHISA5(1485) | BRD3(1567) | NUDCD3(1570) |  |  |  |  |
| NLRP2 | 0.599 | PLXDC2(666) | BRD3(698) | KALRN(960) | NOL10(1006) | CSMD1(1016) | SHISA5(1173) | A2BP1(1603) | IGF1R(1666) | OSBPL1A(1930) | CRIM1(1972) |  |  |  |
| NUDCD3 | 0.616 | NOL10(638) | TMEM180(686) | SVOP(801) | GTDC1(934) | PLXDC2(1188) | CCDC85A(1276) | C1orf21(1534) |  |  |  |  |  |  |
| PLXDC2 | 0.622 | NOL10(540) | SVOP(611) | GTDC1(927) | TMEM180(931) | NUDCD3(1172) | CRIM1(1198) | CCDC85A(1466) | C1orf21(1585) | FAM62B(1695) | BRD3(1896) |  |  |  |
| SVOP | 0.623 | NOL10(627) | PLXDC2(754) | TMEM180(764) | GTDC1(840) | NUDCD3(883) | C1orf21(1120) | CCDC85A(1171) | FAM62B(1347) |  |  |  |  |  |
| DGKH | 0.633 | PLXDC2(395) | NOL10(529) | GTDC1(834) | BRD3(1148) | A2BP1(1293) | SVOP(1356) | NUDCD3(1453) | CCDC85A(1561) | PIB5PA(1610) | TMEM180(1795) |  |  |  |
| OSBPL1A | 0.636 | SVOP(290) | NOL10(501) | PLXDC2(728) | NUDCD3(933) | BRD3(1062) | PIB5PA(1336) | GTDC1(1490) |  |  |  |  |  |  |
| NCAPG2 | 0.660 | TMEM180(297) | NUDCD3(384) | NOL10(513) | BRD3(978) | PLXDC2(1547) |  |  |  |  |  |  |  |  |
| GTDC1 | 0.678 | SVOP(647) | NOL10(696) | NUDCD3(757) | PLXDC2(771) | CCDC85A(1239) | PIB5PA(1499) | C1orf21(1505) | BRD3(1550) | FAM62B(1621) | TMEM180(1819) |  |  |  |
| CCDC85A | 0.705 | C1orf21(360) | TMEM180(749) | SVOP(1474) | NUDCD3(1627) | PLXDC2(1760) | NOL10(1902) |  |  |  |  |  |  |  |
| LRRC16A | 0.728 | NOL10(522) | PLXDC2(576) | NUDCD3(1127) | SVOP(1145) | GTDC1(1285) | CCDC85A(1354) | BRD3(1810) | C1orf21(1827) | SYNPO2(1912) |  |  |  |  |
| C1orf21 | 0.751 | TMEM180(502) | CCDC85A(595) | SVOP(1658) | NOL10(1941) |  |  |  |  |  |  |  |  |  |
| SUFU | 0.753 | BRD3(313) | NOL10(821) | PLXDC2(845) | SYNPO2(1143) | CRIM1(1203) | PIB5PA(1811) |  |  |  |  |  |  |  |
| CPM | 0.792 | PLXDC2(244) | PIB5PA(857) | NOL10(1038) | BRD3(1373) | LAMA1(1677) |  |  |  |  |  |  |  |  |
| GPR179 | 0.808 | hCG_18290(482) | TMEM180(553) | NOL10(801) | SVOP(1278) |  |  |  |  |  |  |  |  |  |
| BRD3 | 0.816 | NOL10(150) | PLXDC2(1389) | NUDCD3(1850) | GTDC1(1952) |  |  |  |  |  |  |  |  |  |
| SHISA5 | 0.819 | TMEM180(583) | PLXDC2(895) | NOL10(1161) | NUDCD3(1183) | BRD3(1186) | C1orf21(1648) |  |  |  |  |  |  |  |
| TMEM180 | 0.829 | C1orf21(583) | NOL10(1123) | CCDC85A(1240) | SVOP(1386) | NUDCD3(1408) | PLXDC2(1864) |  |  |  |  |  |  |  |
| hCG_18290 | 0.831 | hCG_18290(5) | NOL10(656) | NUDCD3(707) | TMEM180(968) | SVOP(1214) | PLXDC2(1595) | GPR179(1688) | CCDC85A(1877) |  |  |  |  |  |
| DNAJA1 | 0.849 | BRD3(445) | NOL10(728) | PLXDC2(1234) | SHISA5(1685) | TMEM180(1831) | CRIM1(1881) |  |  |  |  |  |  |  |
| NOL10 | 0.882 | SVOP(1106) | TMEM180(1149) | PLXDC2(1194) | BRD3(1257) | NUDCD3(1292) | GTDC1(1581) | FAM62B(1897) |  |  |  |  |  |  |
| ZNF71 | 0.926 | C1orf21(976) | NOL10(1286) | TMEM180(1427) | PLXDC2(1734) |  |  |  |  |  |  |  |  |  |

Ten clusters of related genes with GRAIL *P*-values < 0.05 are generated out of the 63 gene clusters using the GRAIL package.
